# Efficient Tetracycline Adsorption by KOH-Activated Yak-Dung Biochar: Mechanistic Insights

**DOI:** 10.3390/ma18245591

**Published:** 2025-12-12

**Authors:** Wei Li, Yi Wu, Zengrui Gu, Chen Yang, Jiacheng Song, Jiaxuan Li, Yahao Zhang, Xuebin Lu, Jian Xiong

**Affiliations:** 1 School of Ecology and Environment, Xizang University, Lhasa 850012, China; weili_xzdx@163.com (W.L.); yiw_0000@126.com (Y.W.); gwg02108@163.com (Z.G.); yang-chen02468@126.com (C.Y.); songjiacheng1031@163.com (J.S.); ljxx@tju.edu.cn (J.L.); 13228904532@163.com (Y.Z.); 2 School of Environmental Science and Engineering, Tianjin University, Tianjin 300350, China; 3 Henan Key Laboratory of Water Pollution Control and Rehabilitation Technology, Henan University of Urban Construction, Pingdingshan 467036, China

**Keywords:** tetracycline hydrochloride, yak dung, biochar, modified, adsorption

## Abstract

In this study, yak dung was used as a precursor to prepare biochar (KYBC600) via high-temperature pyrolysis and KOH modification for the adsorption of tetracycline hydrochloride (TCH) from aqueous solution. Compared with commonly used biomass feedstocks such as straw and fruit shells, the resource-oriented utilization of yak dung holds dual significance: it contributes to regional environmental management and enables high-value conversion of waste, underscoring its distinct regional relevance and potential for synergistic environmental governance. The physicochemical properties of KYBC600 were characterized using BET surface area analysis, FTIR, XRD, and SEM. Adsorption behavior and mechanisms were systematically investigated through kinetic, isotherm, and thermodynamic studies, supplemented by response surface methodology (RSM). The results demonstrated that the adsorption of TCH onto KYBC600 followed pseudo-second-order kinetics and the Langmuir model, with a maximum adsorption capacity of 54.10 mg·g^−1^. Multiple synergistic mechanisms governed the adsorption process, including pore filling, π–π stacking, electrostatic interactions, hydrogen bonding, and complexation with Ca^2+^, with chemical adsorption playing a dominant role. KYBC600 demonstrated excellent adsorption performance and regeneration capability across a wide pH range and in various real water matrices. This study provides novel perspectives on the resource utilization of livestock waste in plateau regions and provides a technical reference for treating low-concentration TCH-containing wastewater.

## 1. Introduction

Tetracycline hydrochloride (TCH) is a typical antibiotic widely employed in medical and animal husbandry sectors [1]. However, as TCH cannot be completely metabolized in humans and animals, it enters the environment via excretion [2], leading to continuous accumulation of this biotoxin. TCH residues are frequently detected in various environmental compartments [3], posing potential threats to human health and ecological safety. TCH exhibits relatively high lipophilicity, which promotes its bioaccumulation and potential toxic effects in organisms [4]. Meanwhile, the persistence of TCH in the environment may foster the emergence of antibiotic resistance genes (ARGs). These genes can spread among pathogenic bacteria, making them more difficult to eradicate [5]. Notably, TCH concentrations in natural waters are generally low, yet due to its antibacterial nature, conventional water treatment processes (e.g., activated sludge systems) exhibit limited effectiveness in removing TCH [6,7].

Current monitoring data indicate variations in TCH residual concentrations across different water bodies: tetracycline antibiotics are often among the categories detected at the highest concentrations in lakes and reservoirs [8]; the total concentration of three tetracycline drugs in karst groundwater in southwestern China reached 421 ng·L^−1^ [9]; total antibiotic levels (including tetracyclines) in groundwater in Harbin ranged from 0.02 to 612 ng·L^−1^ [10]; and the average tetracycline concentration in surface water of the Yellow River Basin was 219.36 ng·L^−1^ [11]. According to the Chinese Standards for Drinking Water Quality (T/CPMA 022-2020), the minimum detection concentration for tetracycline antibiotics can reach 1.56–4.98 ng·L^−1^ [12]. Conventional processes such as the activated sludge method show limited removal efficiency for TCH, primarily relying on adsorption and transfer rather than degradation mechanisms. Moreover, the stable structure and low biodegradability of TCH increase the risk of secondary pollution during treatment [13]. Consequently, various efficient alternative technologies have been developed for TCH degradation, including photocatalysis [14], Fenton/photo-Fenton reactions [15], persulfate activation [16], catalytic ozonation [17], catalytic membrane technology [18], and micro-nano bubble-enhanced advanced oxidation processes [19]. These methods primarily achieve efficient TCH decomposition through free radical-based reactions. It is particularly noteworthy that TCH exposure can induce antibiotic resistance, ecotoxicity, and health hazards such as allergic reactions, liver damage, and intestinal microbiota disruption [20], underscoring the urgency of its control. Currently, both international and Chinese regulations have established a series of requirements for antibiotic use and discharge, such as the “Guidelines for the Use of Antibiotic Drugs” and “Restrictions on Veterinary Drug Use”. Nevertheless, the management of antibiotic residues in the environment still relies predominantly on technological treatment approaches [21].

Current strategies for TCH removal from aquatic environments include adsorption, advanced oxidation, membrane separation, and electrochemical technologies [22]. Although advanced oxidation and membrane processes achieve high removal efficiencies, their substantial energy consumption and high operational costs restrict large-scale application [23,24,25]. In contrast, adsorption has gained extensive attention as a cost-effective, environmentally friendly, operationally simple, and versatile alternative [26].

Biochar is a carbon-rich, highly aromatic, and stable porous solid produced from the thermochemical conversion of biomass under oxygen-limited conditions at high temperatures [27,28,29]. Its feedstocks are diverse and widely available, including crop straws, fruit shells, municipal sludge, and livestock manure [30,31]. Biochar typically possesses a high specific surface area, well-developed pore structure, and abundant oxygen-containing surface functional groups, which collectively contribute to its remarkable adsorption performance [32]. Due to its simplicity of operation, high cost-effectiveness and environmental friendliness, biochar has emerged as a highly promising material for water environment remediation [33].

Notably, pristine biochar often suffers from limitations in pore structure and surface functional groups. Commonly employed modification methods include acid treatment, alkaline activation, metal impregnation, and ball milling [34]. These modification strategies aim to optimise the surface structure of biochar, increase the number of oxygen-containing functional groups, enhance specific surface area, and improve stability, thereby enhancing its adsorption performance. For instance, Nie et al. [35] reported that acid-modified biochar exhibited a larger specific surface area (338.6 m^2^·g^−1^) and more oxygen-containing functional groups than unmodified biochar, along with stable adsorption performance over a wide pH range.

Conventional modification strategies for biochar include acid treatment, alkaline activation, metal (ion or oxide) impregnation, and ball-milling [36]. Among various modification strategies, alkaline activation has proven particularly effective in improving biochar’s adsorption performance. Potassium hydroxide (KOH) is a widely used activating agent that significantly increases specific surface area and optimizes pore structurer [37]. Shi et al. [38] demonstrated that KOH-activated walnut shell biochar pyrolyzed at 900 °C (KWS900) showed a substantially increased BET surface area and achieved a maximum tetracycline (TC) adsorption capacity of 607.00 ± 31.87 mg·g^−1^, while maintaining high stability and reusability across a broad pH range, even in the presence of coexisting ions. Similarly, Qin et al. [39] also found that KOH modification significantly increased the specific surface area (1531 m^2^·g^−1^) and the number of surface functional groups of biochar, notably enhancing its adsorption capacity for TCH.

On the Qinghai–Tibet Plateau, where animal husbandry is relatively developed, yak dung is partially used as fuel by local residents but is often indiscriminately piled around pastures or water sources. Given that yak dung may contain certain microbial pathogens [40], such practices not only pose risks to human health but also represent a waste of agricultural and pastoral resources. Converting yak dung into biochar via pyrolysis for the adsorption and removal of TCH from water aligns with the circular management concept of “treating waste with waste”, offering a viable pathway for regional pollution control and high-value waste utilization. This study employs high-temperature pyrolysis combined with KOH modification to prepare biochar from yak dung, with the aim of providing technical support for the resource utilization of plateau livestock waste and the treatment of low-concentration TCH wastewater.

## 2. Materials and Methods

### 2.1. Materials and Equipment

#### 2.1.1. Experimental Instrumentation

The following analytical and preparative instruments were employed throughout the experimental campaign: a forced-air drying oven (Model DHG-9055A, Shanghai Yiheng Scientific Instruments Co., Ltd., Shanghai, China), a thermostatic water-bath shaker (Model SHA-CA, Beijing Yongguangming Medical Instrument Co., Ltd., Beijing, China), an electronic analytical balance (Model GL2004B, Shanghai Youke Instrument Co., Ltd., Shanghai, China), a diaphragm vacuum pump (Model SCJ-10, Shanghai Huichuang Chemical Instruments Co., Ltd., Shanghai, China), a UV–Vis spectrophotometer (SPECORD 50 PLUS, Analytik Jena AG, Jena, Germany), a micro benchtop tube furnace (Model TFH-1200-50-200, Anhui Kemi Instrument Co., Ltd., Hefei, China), a Fourier-transform infrared spectrometer (Model Great20, Zhongkeruijie (Tianjin) Technology Co., Ltd., Tianjin, China), a specific surface area and porosity analyzer (Model V-Sorb 2800TP, Beijing Guoyi Jingce Technology Co., Ltd., Beijing, China), an X-ray diffractometer (Model TZL-30, Dandong Tongda Technology Co., Ltd., Dandong, China), a field-emission scanning electron microscope (Model SEM5000, Quantum Design China, Hefei, China), a magnetic stirrer (Model 85-1, Shanghai Sile Instrument Co., Ltd., Shanghai, China), a circulating water-jet vacuum pump (Model SHZ-D (III), Shanghai Lichen-Bangxi Instrument Co., Ltd., Shanghai, China), a hydraulic press (Model PP-15, Zhongkeruijie (Tianjin) Technology Co., Ltd., Tianjin, China), a high-speed universal pulverizer (Model FW-100, Beijing Yongguangming Medical Instrument Co., Ltd., Beijing, China), a numerical-control ultrasonic cleaner (Model SJ-QXJ-4, Tianjin Shunji Technology Co., Ltd., Tianjin, China), a laboratory ultrapure water system (Model AKSJL-RO-C2, Jinan Aiken Environmental Protection Technology Co., Ltd., Jinan, China), a Malvern Zeta Potential Analyzer (Software version 8.01.4906, Malvern Instruments Ltd., Malvern, UK) and using an X-ray photoelectron spectrometer (Model 250Xi, Thermo Fisher Scientific, Waltham, MA, USA).

#### 2.1.2. Experimental Reagents

The principal reagents utilized throughout the experimental procedures were as follows: tetracycline hydrochloride (TCH, purity ≥ 98.0%, Hefei Qiansheng Biotechnology Co., Ltd., Hefei, China), potassium hydroxide (analytical grade, Sichuan Xilong Scientific Co., Ltd., Chengdu, China), sodium hydroxide (technical grade, purity 96.0%, Shandong Keyuan Biochemical Co., Ltd., Jinan, China), hydrochloric acid (analytical grade, Shanghai Aladdin Biochemical Technology Co., Ltd., Shanghai, China), sodium chloride (analytical grade, Chengdu Kelong Chemical Co., Ltd., Chengdu, China), and potassium bromide (for spectroscopy, purity ≥ 99.5%, Tianjin Nuolei Xinda Technology Co., Ltd., Tianjin, China).

### 2.2. Biochar Preparation

#### 2.2.1. Preparation of Pristine Biochar

**Figure 1:**
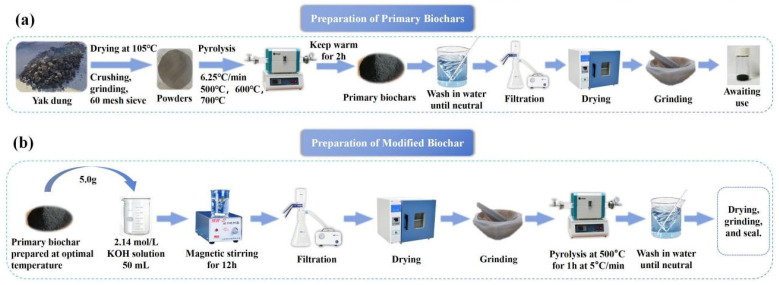
Biochar preparation process. (**a**) Preparation process of primary biochar; (**b**) Preparation process of KOH-modified biochar. All operations were performed at room temperature.

Raw biochar was prepared through one-step pyrolysis of naturally air-dried yak dung in a tubular furnace. The dung was collected from a pastoral village in Maizhokunggar County, Lhasa City, and delivered to the laboratory within 24 h. As shown in Figure 1a, the feedstock was first sun-dried for 10 h to remove moisture and extraneous matter, oven-dried, crushed, ground, and sieved to <250 μm (60 mesh). An accurately weighed aliquot (~5 g) was placed in a quartz boat and pyrolyzed under a continuous N_2_ flow at target temperatures of 500, 600, or 700 °C (heating rate: 6.25 °C·min^−1^; residence time: 2 h). The resulting products are referred to as YBC500, YBC600, and YBC700, respectively. All biochar samples were thoroughly rinsed with deionized water until the effluent reached neutral pH, filtered, dried at 105 °C, and lightly ground before use. The optimal pyrolysis temperature was determined based on comprehensive characterization and comparative adsorption tests performed under identical conditions; the biochar demonstrating the highest removal efficiency was selected for subsequent modification and mechanism investigation.

#### 2.2.2. Preparation of Modified Biochar

The preparation procedure for KOH-modified biochar is schematically illustrated in Figure 1b. A 5.0 g aliquot of the pristine biochar (YBC600) was transferred into a 100 mL glass beaker and impregnated with 50 mL of 2.14 mol·L^−1^ KOH solution. The beaker was sealed with Parafilm and magnetically stirred for 12 h at ambient temperature. The mixture was then subjected to suction filtration using a 0.45 μm membrane. The solid residue was dried at 105 °C for 12 h to obtain KOH-impregnated biochar. The dried product was ground into fine powder, placed in a quartz boat, and heated in a tube furnace at 600 °C under N_2_ atmosphere for 1 h with a heating rate of 6.25 °C·min^−1^, yielding KOH-modified yak dung biochar, denoted as KYBC. The KYBC was repeatedly washed with deionized water until the filtrate reached neutral pH, as determined by universal pH test paper. The sample was subsequently dried in an oven at 105 °C for 12 h, ground again, sieved to obtain a powdered form, and stored in a sealed container for further use. The modification temperature of 600 °C was selected based on comprehensive considerations including specific surface area, pore structure, type and quantity of surface functional groups, and preliminary adsorption experimental results.

### 2.3. Material Characterization

Both the raw biochar samples prepared at different pyrolysis temperatures and the modified biochar samples were characterized by Fourier transform infrared spectroscopy (FTIR), X-ray diffraction (XRD), scanning electron microscopy (SEM), and specific surface area and pore size distribution analysis (BET) and Characterization by X-ray photoelectron spectroscopy (XPS).

- Scanning electron microscopy (SEM) observations were carried out on an SEM5000 scanning electron microscope using an accelerating voltage of 2 kV, beam current mode of 20 μm, resolution of 3072 × 2048 pixels, dwell time of 10 μs, and emission current of 109.75 μA.
- Fourier transform infrared (FTIR) spectra were acquired on a Great20 spectrometer with a scanning range of 500–4000 cm^−1^, resolution of 4 cm^−1^, and 32 accumulated scans.
- X-ray diffraction (XRD) phase analysis was conducted on a TZL-30 X-ray diffractometer equipped with Cu Kα radiation, operated at 30 kV and 20 mA, with a scanning range of 5–85° (2θ), a step size of 0.0414°, and a sampling time of 0.5 s per step.
- The specific surface area and pore structure were determined with a V-Sorb 2800TP analyzer using nitrogen as the adsorbate and helium as the carrier gas. Adsorption–desorption isotherms were measured at 77 K (liquid nitrogen temperature), following sample pretreatment at 120 °C for 30 min.
- TCH solution absorbance was measured at a wavelength of 357 nm using a SPECORD 50 PLUS UV–visible spectrophotometer.
- The Zeta potential of the biochar was measured using a Malvern Zeta potential analyzer (Ver. 8.01.4906).

X-ray photoelectron spectroscopy (XPS) analysis was performed using a Thermo Scientific ESCALAB 250Xi spectrometer (Thermo Fisher Scientific, Waltham, MA, USA).X-ray photoelectron spectroscopy (XPS) characterization was performed using a Thermo Scientific Escalab 250Xi spectrometer with a monochromatic Al Kα X-ray source (hν = 1486.6 eV). The measurements were conducted with a power of 150 W, a beam spot size of 650 μm, an accelerating voltage of 14.8 kV, and a filament current of 1.6 A. The binding energy scale was calibrated by setting the adventitious carbon C 1s peak to 284.8 eV. Survey spectra were acquired with a pass energy of 100 eV and a step size of 1 eV, while high-resolution narrow scans were recorded with a pass energy of 20 eV and a step size of 0.1 eV.

### 2.4. Adsorption Experiments of Biochar for Tetracycline Hydrochloride (TCH) in Aqueous Solution

#### 2.4.1. Calibration Curve for Tetracycline Hydrochloride (TCH)

**Figure 2:**
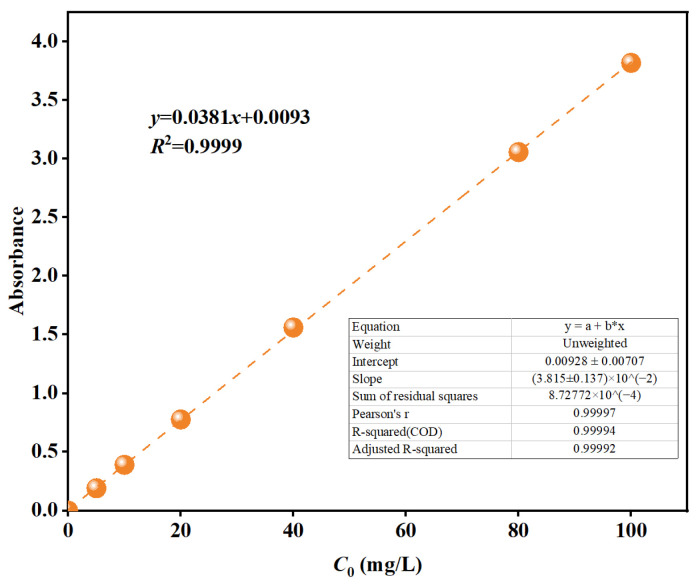
Calibration curve of tetracycline hydrochloride (measured at room temperature): y = 0.0381x + 0.0093 (R^2^ = 0.9999).

A 10.0 mg sample of tetracycline hydrochloride (TCH) was weighed into a 100 mL beaker. Dissolution was achieved with continuous stirring using a glass rod. The solution was then transferred to a 100 mL volumetric flask and diluted to volume, followed by thorough mixing to prepare a 100 mg·L^−1^ TCH stock solution. The stock solution was diluted to concentrations of 5, 10, 20, 40, and 80 mg·L^−1^. Full-wavelength scanning of the TCH solutions was performed using a UV-Vis spectrophotometer, identifying the maximum absorption wavelength at 357 nm. Absorbance values of the different TCH concentrations were measured at 357 nm [38]. A standard calibration curve was constructed. The TCH standard curve is shown in Figure 2. Linear fitting using Origin software (version 9.14) yielded the standard working curve for TCH, with the regression equation y = 0.0381x + 0.0093 and a correlation coefficient R^2^ = 0.9999.

#### 2.4.2. Screening of Pristine Biochar at Different Pyrolysis Temperatures

The pristine biochar prepared at different temperatures were compared using XRD, FTIR, SEM, and BET characterization. Based on these analyses, the biochar demonstrating superior TCH adsorption performance was selected for subsequent modification. Adsorption experiments were conducted under identical conditions (reaction time: 6 h, temperature: 25 °C, initial TCH concentration: 100 mg·L^−1^, biochar dosage: 1.5 g·L^−1^) to evaluate TCH removal. For each test, the biochar sample was added to a 50 mL centrifuge tube containing 20 mL of TCH solution. Adsorption was carried out in a constant-temperature water bath shaker at 150 rpm in the dark. After shaking, the supernatant was collected, filtered through a 0.45 μm membrane, and the absorbance of the filtrate was measured. All experiments were performed in triplicate, and average values were calculated.

#### 2.4.3. Single-Factor Experiments on TCH Adsorption by Biochar

All experiments used ultrapure water. The adsorbents were the optimal pristine biochar and its modified counterpart, both prepared at the selected pyrolysis temperature. The effects of adsorption time, adsorbent dosage, initial TCH concentration, and initial solution pH on TCH adsorption were investigated separately. Adsorption kinetics, intraparticle diffusion, thermodynamics, and isotherms were analyzed.

The adsorption time study spanned a range of 0 to 420 min, conducted at 150 rpm and 25 °C, with a TCH concentration of 20 mg·L^−1^. Adsorption experiments were performed using dilutions of the stock solution to varying initial TCH concentrations (5 mg·L^−1^ to 100 mg·L^−1^). NaCl solutions (0–1.0 mol·L^−1^) were added as co-existing ions to 20 mg·L^−1^ TCH solutions to investigate their effect on TCH removal by modified biochar. All experiments were conducted in triplicate.

For adsorption kinetics, biochar was added to 20 mL of 20 mg·L^−1^ TCH under the optimal dosage and without ions. Adsorption isotherms were obtained by adding 10 mg of adsorbent to 20 mL TCH solutions (5–100 mg·L^−1^) at 25 °C. Thermodynamic studies were performed at 15, 25, and 35 °C. All shaking experiments were conducted at 150 rpm in the dark using a horizontal constant-temperature shaker. After adsorption, 10 mL of supernatant was sampled with a syringe, filtered through a 0.45 μm membrane, and the TCH concentration was determined by measuring absorbance at 357 nm with a UV-Vis spectrophotometer.

#### 2.4.4. Response Surface Methodology (RSM) Optimization of TCH Adsorption Conditions by KOH-Modified Biochar

Based on single-factor experiments, a Box–Behnken central composite design was employed for response surface optimization. Adsorption time, biochar dosage, and initial solution pH—identified as significant factors affecting TCH removal efficiency—were selected as independent variables. With TCH removal efficiency as the response, a three-factor, three-level experimental design was implemented to investigate interactive effects and determine the optimal adsorption conditions.

During KOH modification, biochar was mixed thoroughly with the KOH solution in a 250 mL beaker and stirred using a magnetic stirrer. The stirring speed was set to position “2” (note: the equipment lacks an RPM display, so exact rotational speed cannot be specified). After modification, vacuum filtration was performed at −0.08 MPa using a 0.45 μm membrane to achieve solid–liquid separation.

#### 2.4.5. Adsorption–Desorption Regeneration Performance of KOH-Modified Biochar

Based on the single-factor adsorption experimental results and referencing the ultrasonic method in reference [41], the reusability of modified biochar was investigated. Triplicates were performed per cycle, wherein 30 mg of modified biochar (KYBC) was introduced into a 50 mL centrifuge tube containing 20 mL of 20 mg·L^−1^ TCH solution, subjected to light-proof shaking at 150 rpm and 25 °C for 6 h in a horizontal thermostatic shaker; the resulting suspension was then filtered through a 0.45 µm membrane, the TCH concentration in the filtrate was quantified, while the biochar retained on the membrane was collected into a fresh 50 mL centrifuge tube, mixed with 30 mL of ultrapure water, and ultrasonicated (operating parameters: power supply 220 V/50 Hz, ultrasonic frequency 40 kHz, ultrasonic power 100 W, heating power 500 W, heating temperature set at 30 °C) for 2 h to desorb TCH; the post-sonication suspension was filtered through a 0.45 µm membrane, the recovered biochar was dried to constant weight, and subsequently reused to initiate the subsequent adsorption cycle, with this adsorption–desorption sequence repeated five times to evaluate the recyclability of KYBC.

#### 2.4.6. Adsorption of TCH by KOH-Modified Biochar in Different Water Matrices

A 100 mL water sample from the campus artificial lake (Siyuan Lake) was filtered to remove debris and spiked with TCH to 20 mg·L^−1^. Similarly, 100 mL aliquots of laboratory tap water and ultrapure water were separately spiked with TCH to 20 mg·L^−1^ to simulate contaminated aqueous environments. Under the response surface-optimized adsorption conditions, modified biochar KYBC600 was added to each of the three water samples in 50 mL centrifuge tubes and shaken at 25 ± 1 °C for 7 h in the dark. After shaking, the supernatant was filtered, and its absorbance was measured to compare TCH removal efficiencies across different water matrices. All experiments were conducted in triplicate, and mean values were calculated.

### 2.5. Mathematical Models

#### 2.5.1. Removal Efficiency and Adsorption Capacity

The biochar yield, denoted as *φ* (%), was calculated according to Equation (1).

(1) $$\varphi =\frac{m}{m_{0}}\times 100\%$$

where *m* is the dry mass of the resulting biochar (g) and *m*_0_ is the initial dry mass of the yak dung (g).

The adsorptive performance of the biochar toward tetracycline hydrochloride (TCH) was assessed by two quantitative indices: (1) the percentage removal efficiency, *R* (%), and (2) the equilibrium adsorption capacity, *Q*_e_ (mg·g^−1^).

where

(2) $$R=\frac{C_{0}-C_{i}}{C_{0}}\times 100\%$$

(3) $$Q_{e}=\frac{{(C}_{0}-C_{i})\times V}{m}$$

In these expressions, *C*_0_ is the initial TCH concentration (mg·L^−1^), *Cᵢ* is the equilibrium TCH concentration (mg·L^−1^), *V* is the solution volume (L), and *m* is the mass of the biochar employed (g).

#### 2.5.2. Adsorption Kinetics

In accordance with the experimental data obtained in Section 2.4.3, kinetic analysis was performed by non-linear regression of the pseudo-first-order and pseudo-second-order models using Origin software; the derived kinetic parameters were subsequently employed to appraise the adsorption performance.

1. The pseudo-first-order kinetic model presumes that the overall rate of adsorption is governed by diffusion processes, with the driving force being proportional to the first power of the difference between the equilibrium adsorption capacity and the instantaneous amount adsorbed. The corresponding non-linear rate equation is expressed as
  (4) $$Q_{t}=Q_{e}(1-e^{{-k}_{1}t})$$
  where *Q*_t_ and *Q*_e_ are the amounts of adsorbate (mg·g^−1^) at any time *t* and at equilibrium, respectively, and *k*_1_ is the pseudo-first-order rate constant (min^−1^).
2. The pseudo-second-order kinetic model postulates that the rate-limiting step is chemisorption, involving valence forces through electron sharing or exchange between adsorbate and adsorbent. The corresponding non-linear expression is
  (5) $$Q_{t}=\frac{Q_{e}^{2}k_{2}t}{{1+Q}_{e}k_{2}t}$$
  where Q_t_ and Q_e_ represent the adsorbed amounts (mg·g^−1^) at time t (min) and at equilibrium, respectively, and k_2_ is the pseudo-second-order rate constant (g/mg·min).
3. The intraparticle diffusion model presumes that the adsorption rate is governed by diffusion within the pores of the adsorbent. The corresponding model equation is expressed as
  (6) $$Q_{t}=k_{i}t^{1/2}+C$$
  In the intraparticle diffusion model, Qt (mg·g^−1^) denotes the amount of TCH adsorbed at time t (min), ki [mg (g min^1/2^)^−1^] represents the intraparticle diffusion rate constant, and C is the intercept that reflects the thickness of the boundary layer.

#### 2.5.3. Adsorption Thermodynamics

Thermodynamic parameters governing the adsorption of tetracycline hydrochloride (TCH) onto the biochar were determined at three discrete temperatures (15, 25, and 35 °C). The fundamental relationships are expressed as follows:

(7) $$K=\frac{Q_{e}}{C_{e}}$$

(8) $$lnK=\frac{∆S^{\theta }}{R}-\frac{∆H^{\theta }}{RT}$$

(9) $$∆G^{\theta }=∆H^{\theta }-T∆S^{\theta }=-\mathrm{RT}\ln K$$

The thermodynamic analysis employs *Q_e_* (mg·g^−1^) to quantify the equilibrium uptake of TCH by the biochar and *C_e_* (mg·L^−1^) as the residual solute concentration at equilibrium; the dimensionless equilibrium constant *K* derived from these values is then combined with the absolute temperature *T* (K) and the universal gas constant *R* (8.314 J·mol^−1^·K^−1^) to calculate the Gibbs free-energy change Δ*Gᶿ* (kJ·mol^−1^), whose sign and magnitude reflect adsorption spontaneity, while the complementary enthalpy change Δ*Hᶿ* (kJ·mol^−1^) and entropy change Δ*Sᶿ* (kJ·mol^−1^·K^−1^) elucidate the energetic and disorder contributions driving the process.

#### 2.5.4. Adsorption Isotherms

Adsorption isotherms were acquired at 25 °C and modeled with the Langmuir, Freundlich and Temkin equations, expressed as follows:

(10) $$Q_{e}=\frac{Q_{m}K_{L}C_{e}}{{1+K}_{L}C_{e}}$$

(11) $$Q_{e}=K_{F}C_{e}^{\frac{1}{n}}$$

(12) $$Q_{e}=\frac{RT}{b}\ln K_{T}+\frac{RT}{b}lnC_{e}$$

(13) $$R_{L}=\frac{1}{1+K_{L}C_{0}}$$

*Q*_m_ denotes the theoretical maximum adsorption capacity (mg·g^−1^); *C*_e_ is the equilibrium TCH concentration (mg·L^−1^); *K*_L_ is the Langmuir constant related to adsorption energy (L·mg^−1^); *K_F_* and n are the Freundlich constants reflecting adsorption capacity and intensity, respectively [(mg g^−1^) (mg L^−1^)^−1^/^n^]; *K_T_* is the Temkin equilibrium binding constant (L·g^−1^); *b* is the Temkin constant; *R* is the ideal gas constant (8.314 J·mol^−1^·K^−1^); *T* is the absolute temperature (K); and *R*_L_ is the dimensionless separation factor for the Langmuir isotherm.

### 2.6. Investigation of Adsorption Mechanism of KOH-Modified Biochar for TCH in Water

The adsorption mechanism of tetracycline hydrochloride (TCH) from aqueous solution by the modified biochar was inferred from systematic characterization using FTIR, XRD, SEM, and XPS, by correlating alterations in surface functional groups, crystalline phases, and pore architecture.

## 3. Results and Discussion

### 3.1. Adsorption of TCH in Water by Pristine Biochar Prepared at Different Temperatures

#### 3.1.1. Characterization of Pristine Biochar

The BET characterization results and yields of pristine biochar prepared at different pyrolysis temperatures are summarized in Table 1. As an adsorbent, the specific surface area and pore architecture of biochar are known to markedly govern its adsorption capacity [42]. With increasing pyrolysis temperature, the specific surface area first increases and then decreases, the total pore volume progressively rises, while the average pore diameter decreases initially and subsequently increases. As shown in Table 1, when the pyrolysis temperature increased from 500 °C to 600 °C, the specific surface area of the biochar increased from 8.687 m^2^·g^−1^ to 26.646 m^2^·g^−1^, while the average pore width decreased from 10.166 nm to 6.377 nm, indicating an increase in the proportion of micropores. This change is mainly attributed to the release of volatile organic compounds and the reorganization of the carbon skeleton at moderate temperatures (~600 °C), which promotes micropore formation. However, when the temperature was further increased to 700 °C, the specific surface area decreased to 16.635 m^2^·g^−1^, the average pore width increased to 19.594 nm, and the total pore volume continued to rise to 0.081 cm^3^·g^−1^. This behavior can be explained by the collapse or fusion of some micropores at high temperatures, transforming them into mesopores or macropores, accompanied by further aromatization of the carbon material and growth of graphitic microcrystals. This finding aligns with previous reports by Leng et al. [42].

YBC600, prepared at 600 °C, exhibited a relatively high specific surface area (26.646 m^2^·g^−1^) and a slightly larger pore volume (0.042 cm^3^·g^−1^). Although these absolute values are not exceptionally high, its pore structure is relatively more intact, which may provide a greater number of adsorption sites to a certain extent [43]. In contrast, although YBC700 possesses a higher total pore volume, it is dominated by mesopores and macropores, which contribute limitedly to the adsorption of small-molecule pollutants such as tetracycline. Therefore, based on the BET results, it can be inferred that YBC600 exhibits relatively optimal adsorption potential. It possesses the highest specific surface area and suitable micropore volume among the tested samples, providing more available sites for pollutant adsorption. Furthermore, pyrolysis at this temperature helps preserve oxygen-containing functional groups (e.g., -OH, C=O), which can enhance TCH adsorption via mechanisms such as hydrogen bonding and π–π interactions. Although its specific surface area and pore volume remain moderate, the combination of its textual properties and surface chemistry appears most favorable for adsorption among the three materials. The biochar yield slightly decreased with increasing temperature, from 52.05% at 500 °C to 51.90% at 700 °C, which is attributed to the increased volatilization of components such as cellulose and lipids in yak dung at higher pyrolysis temperatures [44].

**Figure 3:**
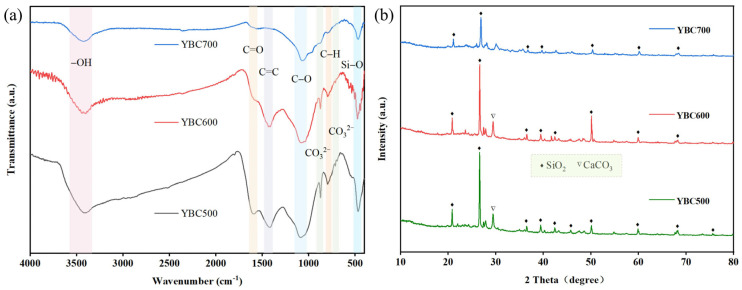
(**a**) FT-IR and (**b**) XRD patterns of yak manure-derived biochar (YBC) prepared at different pyrolysis temperatures (500, 600, and 700 °C). All measurements were conducted at room temperature. FT-IR spectra were recorded in the range of 500–4000 cm^−1^ with a resolution of 4 cm^−1^ and 32 scans. XRD analysis was performed over a 2θ range of 5–85° with a sampling time of 0.5 s, an operating voltage of 30 kV, and a current of 20 mA. The results demonstrate that the raw biochar contains abundant oxygen-containing functional groups, which undergo gradual depletion as the pyrolysis temperature increases.

The FTIR spectrum of YBC is presented in Figure 3a. The absorption band near 470 cm^−1^ is attributed to the bending vibration of Si–O [45], confirming the presence of SiO_2_ and silicate phases, in agreement with the XRD observations. The band at approximately 790 cm^−1^ corresponds to the out-of-plane bending vibration of aromatic C–H [46], whereas the peaks around 712 and 877 cm^−1^ are characteristic of the ${\mathrm{CO}}_{3}^{2-}$ stretching modes in calcite (CaCO_3_) [47], also in agreement with XRD results. The feature near 1100 cm^−1^ originates from C–O stretching [48], and the vibration near 1420 cm^−1^ is assigned to the C=C stretching of aromatic rings [49]. The absorption at ~1590 cm^−1^ is ascribed to C=O stretching [50]. A broad band centered at 3410 cm^−1^ reflects the O–H stretching vibration [51], indicating abundant oxygen-containing functional groups on the YBC surface that can provide active sites for TCH adsorption.

With increasing pyrolysis temperature, the three biochar samples exhibited similar infrared absorption band positions, suggesting comparable types of surface functional groups. This similarity can be attributed to the relatively stable chemical composition of the raw material and the continuous evolution of functional groups during pyrolysis. In the case of YBC700, the intensity of peaks corresponding to -OH, C=O/C=C, and aromatic C-H decreased [52], likely due to the decomposition of oxygen-containing functional groups and further condensation of aromatic structures under high-temperature conditions. Meanwhile, the characteristic peak of ${\mathrm{CO}}_{3}^{2-}$ almost disappeared, which was consistent with XRD results showing no detection of calcite (CaCO_3_), indicating thermal decomposition of carbonate minerals at this temperature. In contrast, biochar produced at 600 °C retained relatively abundant and diverse functional groups, providing more adsorption sites and thereby enhancing adsorption efficiency in subsequent experiments.

The XRD patterns of YBC are displayed in Figure 3b. Relatively sharp reflections are observed at 2θ = 20.87°, 26.63°, 36.42°, 39.50°, 42.40°, 50.06°, and 59.87°, all of which can be indexed to quartz (SiO_2_: PDF#46-1045). Additional peaks attributable to calcite (CaCO_3_: PDF#05-0586) are present near 2θ ≈ 29.40° in YBC500 and YBC600, whereas these calcite reflections disappear in YBC700, suggesting that the elevated temperature (700 °C) promotes the conversion of CaCO_3_ into thermally stable phases [53].

**Figure 4:**
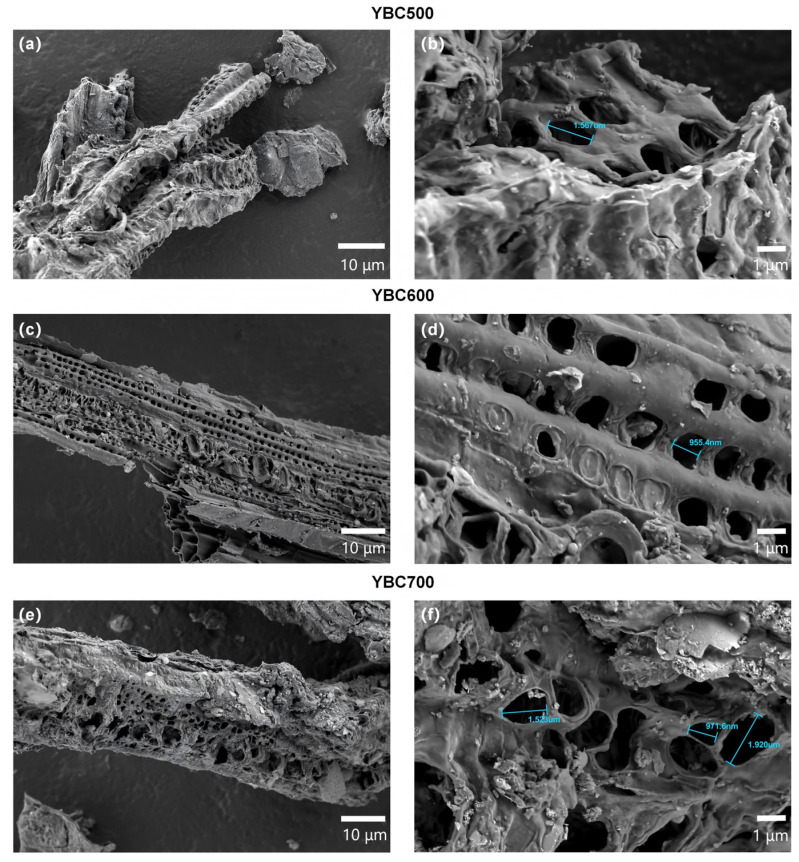
The evolution of the surface pore structure in yak manure-derived biochar (YBC) with increasing pyrolysis temperature is depicted in the SEM images. All images were acquired using a scanning electron microscope operated at an accelerating voltage of 2.0 kV. As shown in panels (**a**,**b**) for YBC500, (**c**,**d**) for YBC600, and (**e**,**f**) for YBC700 at magnifications of 10 µm and 1 µm, respectively, the micrographs reveal that YBC600 exhibits a more uniform and ordered arrangement of surface pores along with a well-preserved structural integrity.

SEM micrographs of YBC are presented in Figure 4. Based on the micrographs, particulate matter was observed adhering to the surfaces of all three biochar types, with well-developed macropores (defined as pores > 50 nm according to IUPAC standards) clearly visible at the 10-µm magnification level. YBC500 presents a relatively smooth surface with stacked lamellar structures, indicating incomplete pyrolysis [54]. In contrast, YBC600 displays a denser and more regularly arranged pore network with intact structural integrity, thereby increasing the accessible surface area for TCH and favoring its removal. YBC700, however, shows irregular, heterogeneous pores of variable diameter, accompanied by partial collapse and localized flocculent textures; such pore loss and deformation are detrimental to adsorption. Compared with YBC600, YBC700 exhibits fewer pores overall, although the remaining pores are enlarged—an observation ascribed to structural collapse of the pore walls at temperatures exceeding a critical threshold [55].

It should be noted that SEM primarily reflects the microporous/macroscopic channel structures of the material. Smaller micropores (<2 nm) and mesopores (2–50 nm) require characterization via gas adsorption isotherms (BET), as shown in Table 1. Although the absolute pore volume of YBC600 is relatively small (0.042 cm^3^·g^−1^), its micropore-dominated pore structure and relatively intact macroscopic porosity provide a certain foundation for TCH adsorption. Based on the comprehensive characterization results, YBC600 exhibits superior specific surface area, pore structure, and abundant surface functional groups compared to other samples, demonstrating potential advantages for TCH adsorption. Therefore, it was selected as the base material for subsequent modification experiments.

#### 3.1.2. Adsorption of TCH in Water by Pristine Biochar

**Figure 5:**
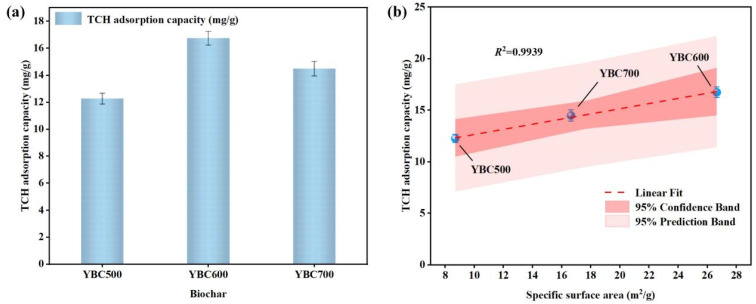
(**a**) Adsorption capacity of pristine biochar for TCH at different pyrolysis temperatures and (**b**) Correlation between the specific surface area of pristine biochar and TCH adsorption capacity. The adsorption experiments were conducted under the following conditions: room temperature, an initial TCH concentration of 100 mg/L, an adsorption time of 6 h, and a biochar dosage of 1.5 g/L. The results demonstrated a positive linear correlation between the specific surface area of the pristine biochar and its tetracycline hydrochloride (TCH) adsorption capacity.

Under the conditions of 25 °C, 100 mg·L^−1^ TCH, 6 h adsorption time, and 1.5 g·L^−1^ biochar dosage, the TCH adsorption results by YBC500, YBC600, and YBC700 are shown in Figure 5a. Under these identical conditions, YBC600 achieved a TCH adsorption capacity of 16.75 mg·g^−1^, superior to YBC500 and YBC700. The linear relationship between the specific surface area of the biochars and their TCH adsorption capacity is shown in Figure 5b. The fitting coefficient R^2^ reached 0.9939, indicating a linear correlation between the specific surface area and the corresponding TCH adsorption capacity for biochars pyrolyzed at different temperatures. This confirms that specific surface area is a key factor influencing the TCH adsorption performance of biochar [56]. An increased specific surface area facilitates the exposure of active sites on the biochar surface [57], thereby promoting the adsorption process. Based on the characterization analysis in Section 3.1.1, YBC600 was selected as the optimal biochar for subsequent experiments.

### 3.2. Adsorption Performance of Biochar and Its KOH Modification for TCH in Water

#### 3.2.1. Characterization of KOH-Modified Biochar

The BET parameters of the KOH-activated biochars are summarized in Table 2. The modified biochar (KYBC600) exhibited a specific surface area of 96.057 m^2^·g^−1^, a total pore volume of 0.101 cm^3^·g^−1^, and an average pore width of 4.212 nm. Compared to YBC600, the modified sample showed an approximately 3.6-fold increase in specific surface area, an improvement in pore volume, and a reduction in average pore width. These changes are primarily attributed to the chemical etching effect of KOH on the carbon framework, which effectively created and opened new micropores [58]. It is noteworthy that although the original biochar YBC600 had a limited pore volume (0.042 cm^3^·g^−1^), its textural properties were significantly enhanced after KOH activation, confirming the necessity of modification for improving adsorption performance.

**Figure 6:**
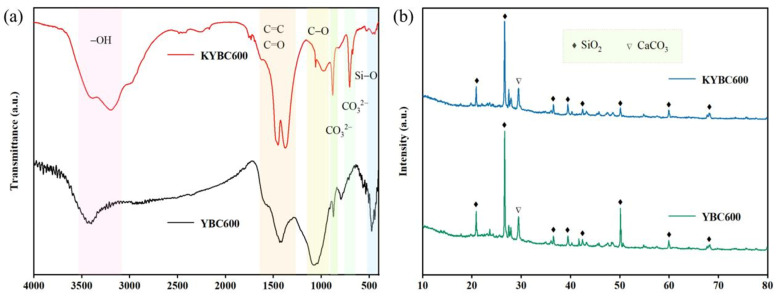
(**a**) FTIR spectra of biochar before and after KOH modification and (**b**) XRD spectra of biochar before and after KOH modification. All characterizations were performed at ambient temperature. Fourier transform infrared (FT-IR) spectroscopy was conducted with a spectral range of 500–4000 cm^−1^, a resolution of 4 cm^−1^, and by averaging 32 scans. X-ray diffraction (XRD) patterns were collected over a 2θ range of 5–85° with a step time of 0.5 s, an operating voltage of 30 kV, and a current of 20 mA. The results demonstrate that KOH modification increased the degree of unsaturation in the biochar, thereby enhancing its aromaticity.

The FTIR spectra before and after modification are shown in Figure 6a. After KOH activation, the -OH stretching vibration peak at approximately 3400 cm^−1^ was significantly intensified, indicating an increase in the content of surface -OH groups [59], which can provide additional adsorption sites. The absorption peaks near 1590 cm^−1^ and 1420 cm^−1^, corresponding to C=C/C=O vibrations, also exhibited notable enhancement, reflecting an increase in aromaticity and unsaturated bonds, likely resulting from the formation of a more developed π-conjugated system π-electron conjugated systems [60,61]. Such structures can interact with aquatic pollutants through π-π stacking [62]. Concurrently, the intensity of the C-O vibration peak (~1100 cm^−1^) weakened and shifted, which is more likely attributed to the transformation of carbon hybridization from sp^3^ to sp^2^, indicating that KOH modification enhances the aromaticity of biochar [63]. After modification, a carbonate-related absorption peak reappeared at approximately 870 cm^−1^, possibly due to the formation of K_2_CO_3_ through chemical reactions between KOH and the biochar surface [64]. The XRD patterns in Figure 6b reveal that the crystalline phases of the biochar remained largely unchanged after modification, with quartz and calcite still being the dominant phases.

**Figure 7:**
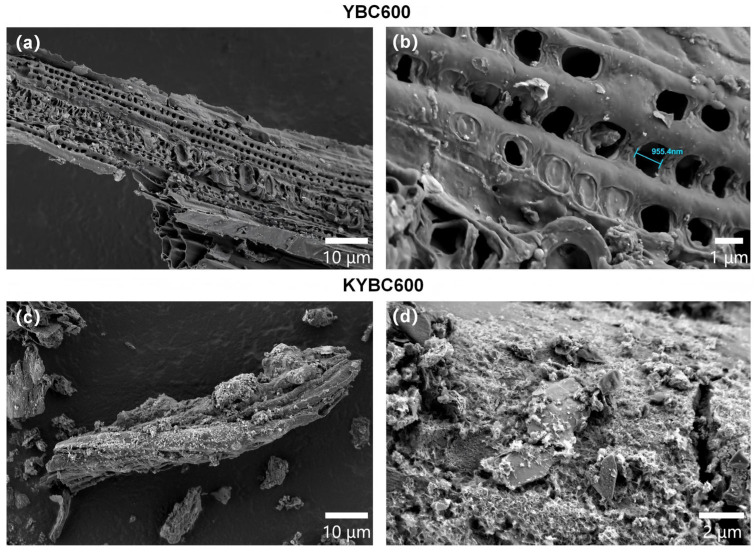
Representative scanning electron microscopy (SEM) images of (**a**,**b**) the pristine biochar (YBC600) and (**c**,**d**) the KOH-modified biochar (KYBC600) are presented. All images were acquired at an accelerating voltage of 2.0 kV. The micrographs reveal that KOH modification induces significant alterations in the surface morphology of the biochar.

SEM images of the biochar before and after modification are presented in Figure 7. After KOH activation, the surface is densely populated with granular deposits, which most likely originate from the etching action of KOH and its subsequent chemical conversion into potassium-containing phases [37]. Simultaneously, the pore architecture exhibits partial disintegration, attributable to the aggressive influence of the KOH reagent [65]. These observations confirm that KOH-impregnated pyrolysis markedly alters the biochar morphology, consistent with the findings reported by Shi et al. [38].

#### 3.2.2. Single-Factor Adsorption Experiments on TCH by Biochar and KOH-Modified Biochar

**Figure 8:**
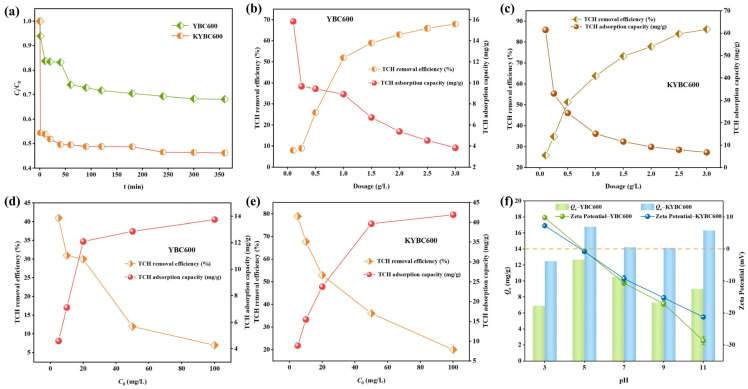
(**a**) TCH adsorption by YBC600 and KYBC600 at different adsorption times; (**b**,**c**) TCH adsorption by biochar at different biochar dosages; (**d**,**e**) TCH adsorption by biochar at different initial TCH concentrations; (**f**) TCH adsorption by biochar and biochar zeta potential at different solution pH values. Reaction conditions: [TCH] = 20 mg·L^−1^, initial pH 5.0, temperature 25 °C. The results indicated that the optimal adsorption conditions for TCH removal by both KYBC600 and YBC600 were as follows: an adsorption time of 6 h, a biochar dosage of 0.5 g/L, an initial TCH concentration of 20 mg/L, and an initial solution pH of 5.

In adsorption studies, adsorption time is a significant factor influencing the contaminant removal efficiency of adsorbents. The TCH adsorption performance of biochars in water at different adsorption times is shown in Figure 8a. The TCH removal efficiency by biochar gradually increased with prolonged adsorption time. Within the initial hour, the TCH adsorption capacity increased rapidly, likely attributable to the abundance of available adsorption sites on the biochar surface during the early adsorption stage [66]. Subsequently, between 1 and 6 h, the adsorption capacity increased slowly and approached near-saturation. At 6 h, the TCH adsorption capacities of YBC600 and KYBC600 reached 11.81 mg·g^−1^ and 20.89 mg·g^−1^, respectively. As the adsorption process proceeded, the adsorption sites on the biochar were progressively occupied by TCH molecules, causing the adsorption rate to gradually decrease until equilibrium was attained [67]. Therefore, considering factors such as time cost, an adsorption time of 6 h was selected for subsequent experiments.

To determine the optimal dosage of the adsorbents, the TCH adsorption performance of YBC600 and KYBC600 under different adsorbent dosages was investigated, as shown in Figure 8b,c. With increasing biochar dosage, the TCH removal efficiency by both YBC600 and KYBC600 improved, whereas their TCH adsorption capacity gradually decreased. It is common practice to select a dosage near the intersection point of contaminant removal efficiency and adsorption capacity as the optimal dosage. At this dosage, a relatively high contaminant removal efficiency and adsorption capacity can be simultaneously achieved, enabling efficient and economical utilization of the adsorbent [68]. Consequently, a biochar dosage of 0.5 g·L^−1^ was selected as the optimal dosage for subsequent experiments.

The influence of the initial TCH concentration on removal efficiency is illustrated in Figure 8d,e. With increasing initial TCH concentration, the percentage removal declines monotonically, whereas the adsorption capacity rises. KOH activation markedly enhances the performance: at an initial TCH concentration of 5 mg·L^−1^, KYBC600 achieves 78.97% removal with an uptake of 8.93 mg·g^−1^, whereas at 100 mg·L^−1^ the removal falls to 20.06% but the capacity increases to 41.95 mg·g^−1^. At low TCH concentrations, adsorption sites are abundant relative to the number of TCH molecules, resulting in high removal efficiencies. Conversely, at elevated concentrations the ratio of TCH molecules to available sites increases; the finite number of active sites becomes rate-limiting, causing the removal efficiency to drop while the adsorption capacity continues to rise [69]. Therefore, an initial TCH concentration of 20 mg·L^−1^ was selected for all subsequent experiments.

Solution pH is a key variable governing biochar–TCH interactions by simultaneously modulating the speciation of TCH and the surface charge of biochar [70]. Adsorption performance was examined across pH 3–11 (Figure 8f). KYBC600 maintained consistently high TCH uptake throughout this pH range, indicating that adsorption is not dominated solely by electrostatic forces but involves significant contributions from chemisorptive interactions [71]. This broad operational pH range enhances its practical utility for applications in diverse aquatic environments.

**Figure 9:**
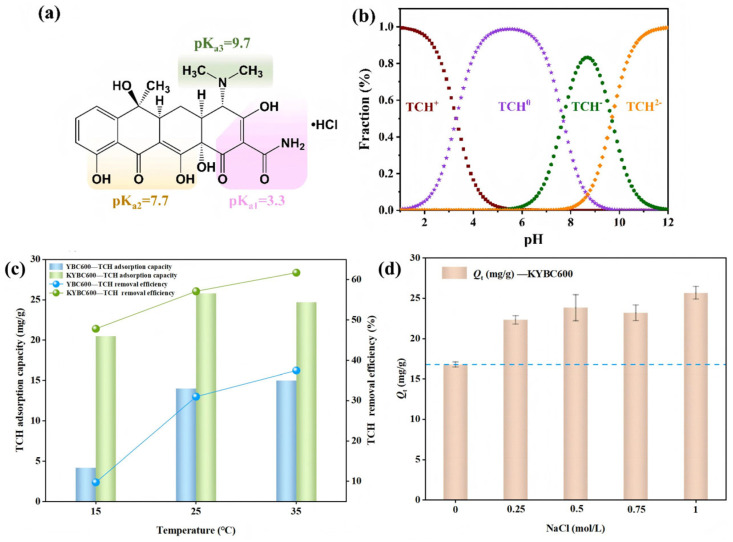
(**a**) Molecular structure of tetracycline hydrochloride [72]; (**b**) Species distribution of tetracycline hydrochloride at different pH values [72]; (**c**) TCH adsorption by biochar at different temperatures; (**d**) Effect of NaCl concentration on TCH adsorption by KYBC600; The blue horizontal dashed line in Figure (**d**) represents the TCH adsorption capacity of biochar KYBC600 without the addition of NaCl. This dashed line is included primarily for comparison purposes, serving as the control group. [TCH] = 20 mg·L^−1^, initial pH 5, temperature 25 °C, dosage 0.5 g·L^−1^. The results demonstrated that the optimal temperature for TCH adsorption by both KYBC600 and YBC600 was 25 °C. Furthermore, the adsorption capacity of KYBC600 for TCH was enhanced with increasing NaCl concentration.

Within the examined pH range, KYBC600 exhibited consistently higher TCH uptake than YBC600. As illustrated in Figure 9a,b, tetracycline hydrochloride (TCH) is amphoteric: below pH 3.3 the dominant species is TCH^+^, whereas above pH 7.7 TCH^−^ or TCH^2−^ prevails.

The lowest adsorption capacity was observed at pH 3, where TCH exists as the cationic TCH^+^ and the biochar surface carries a positive charge (ζ-potential > 0), resulting in electrostatic repulsion [73]. Adsorption reached its maximum as the solution pH approached the biochar’s point of zero charge (pH_PZC_ ≈ 4.85), peaking at pH 5 [38]. Between pH 5 and 7, the neutral TCH^0^ species predominates, implying that hydrogen bonding and π–π interactions are the principal adsorption mechanisms [38]. Further pH increases caused progressively more negative surface charges on the biochar, leading to electrostatic repulsion with anionic TCH species [74]. Consequently, an initial solution pH of 5 was selected for all subsequent experiments.

Temperature significantly influences the adsorption efficiency of contaminants. Figure 9c illustrates the TCH adsorption performance of biochar under different temperature conditions. For KYBC600, the TCH adsorption capacity initially increased with rising temperature but exhibited a decreasing trend at 35 °C, albeit with a minor decline. This reduction may be attributable to TCH decomposition at elevated temperatures [75]. Given that increasing the temperature from 25 °C to 35 °C yielded no substantial enhancement in TCH adsorption performance for the biochar, and considering energy consumption costs, subsequent experiments were conducted at 25 °C.

Real antibiotic wastewaters contain coexisting inorganic ions that can interfere with the adsorption of target pollutants. To evaluate this effect, NaCl was employed as a representative electrolyte. The influence of NaCl concentration on TCH adsorption by modified biochar (KYBC600) is presented in Figure 9d.

Upon the addition of NaCl solutions at varying concentrations, the TCH adsorption capacity of KYBC600 did not decrease; instead, it exhibited varying degrees of increase. The primary reason may be that both TCH and NaCl are chlorides. The presence of the common-ion effect upon NaCl addition reduced the solubility of TCH in water (salting-out effect). Furthermore, TCH molecules inherently possess structures such as aromatic rings and methyl groups, conferring a degree of hydrophobicity. The salting-out effect enhanced the hydrophobicity of TCH, promoting the transfer and diffusion of TCH molecules from the liquid phase to the biochar surface [76]. Consequently, the equilibrium adsorption capacity of TCH onto KYBC600 increased. These results indicate that KYBC600 demonstrates certain advantages and potential in resisting ionic interference in complex real-water environments.

#### 3.2.3. Adsorption Kinetics Analysis

**Figure 10:**
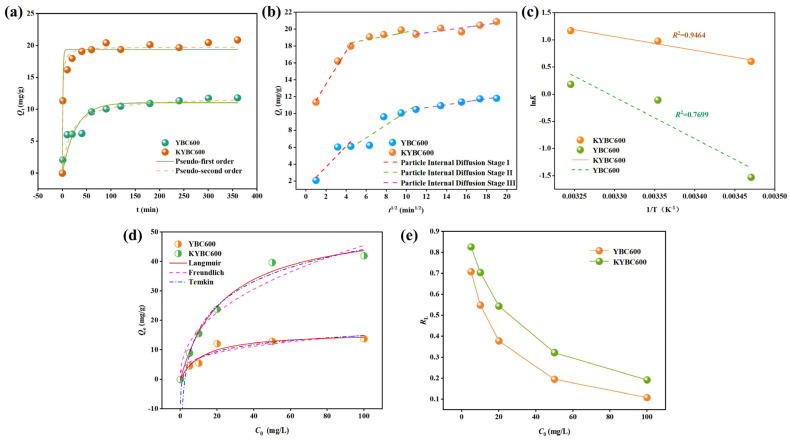
(**a**) Kinetic fitting of TCH adsorption onto biochars KYBC600 and YBC600; (**b**) Fitting curves of the intra-particle diffusion model; (**c**) Linear relationship between lnK and 1/T; (**d**) Isotherm fitting for TCH adsorption onto KYBC600 and YBC600; (**e**) Values of the separation factor (RL). All experiments were conducted under the following conditions: [TCH] = 20 mg·L^−1^, initial pH = 5, and adsorption time t = 6 h. The results demonstrate that the adsorption process is better described by the pseudo-second-order kinetic model, indicating that chemical adsorption is the predominant mechanism. Furthermore, intra-particle diffusion is not the sole rate-limiting step, but rather, the overall process is governed by a combination of multiple factors.

The adsorption kinetics of TCH onto YBC600 and KYBC600 were analyzed using pseudo-first-order and pseudo-second-order models; the fitted curves are shown in Figure 10a, and corresponding parameters are summarized in Table 3. During the initial 60 min, abundant vacant sites on the biochar surface promoted rapid uptake, resulting in a sharp increase in adsorbed amount. Thereafter, progressive occupation of these sites gradually attenuated the rate, and equilibrium was attained within approximately 300 min [65]. Compared to YBC600, KYBC600 exhibited a 3.39-fold higher equilibrium capacity, attributable to its larger specific surface area and well-developed porosity, which provide additional binding sites for TCH.

As shown in Table 3, the pseudo-second-order kinetic model demonstrated a higher goodness-of-fit for both datasets (R^2^ > 0.97), and the theoretical equilibrium adsorption capacity (*Q*_e,cal_) was closer to the experimental values. It should be noted that the pseudo-second-order model is commonly used to describe potential chemical mechanisms in adsorption processes, such as electron sharing or exchange, though it does not exclusively specify the adsorption type [77]. Recent studies have shown that this model may also be applicable to systems dominated by physisorption or adsorption processes involving multiple mechanisms [78]. Combined with FTIR analysis and subsequent mechanistic discussions (e.g., hydrogen bonding, π–π interactions, complexation, etc.), this study infers that chemical interactions contribute significantly to the adsorption of TCH onto KYBC600. This aligns with the mechanistic interpretation reported by Cheng et al. [79] for similar materials adsorbing tetracycline antibiotics, and the adsorption process involves steps such as film diffusion, intraparticle diffusion, and surface adsorption within chemisorption [80].

To investigate the rate-controlling steps in the adsorption process, a particle intraparticle diffusion model was further employed for fitting, with the fitting results shown in Figure 10b and Table 4, respectively. The plots exhibit three distinct linear segments, indicating a three-stage process: (1) external film diffusion of TCH from the bulk solution to the biochar surface, (2) intraparticle diffusion within the biochar pores, and (3) a gradual slowdown of diffusion until adsorption–desorption equilibrium is reached [81]. All fitted curves do not pass through the origin of the coordinates, and the intercepts C_1_, C_2_, and C_3_ are all non-zero, indicating that intraparticle diffusion is not the sole rate-limiting step, and that film diffusion also significantly influences the overall adsorption rate [82]. The rate constants follow the order kᵢ_1_ > kᵢ_2_ > kᵢ_3_, confirming that film diffusion is the primary rate-limiting step in the adsorption of TCH onto biochar [83]. Simultaneously, the boundary layer constant increased in the order C1 < C2 < C3. This indicated that the boundary layer of the biochar exerted distinct levels of influence on the TCH adsorption process. With increasing boundary layer thickness, diffusion resistance progressively increased and the adsorption rate correspondingly decreased, ultimately reaching adsorption–desorption equilibrium in the third stage [84].

#### 3.2.4. Adsorption Thermodynamics Analysis

Thermodynamic analysis is essential for evaluating the spontaneity and energy changes in adsorption processes. However, it is important to note that when calculating thermodynamic parameters using the van’t Hoff equation, the selection of the equilibrium constant (K) critically influences the results. A common error is the inappropriate substitution of the partition coefficient (Q_e_/C_e_) for the thermodynamic equilibrium constant, leading to systematic errors in the calculated values of Δ*G^θ^*, Δ*H^θ^*, and Δ*S^θ^* [85].

In this study, the re-evaluation of adsorption thermodynamics was rigorously conducted by employing the equilibrium constant (*K*_L_) derived from Langmuir model fitting as the thermodynamic equilibrium constant for subsequent parameter calculations.

The relationship between *lnK* and *1/T* was fitted using the experimental data, as depicted in Figure 10c. From the slope and intercept of the resulting straight line, the standard enthalpy change (Δ*H^θ^*) and standard entropy change (Δ*S^θ^*) were calculated; the corresponding thermodynamic parameters are summarized in Table 5. Thermodynamic parameters indicate that for both YBC600 and KYBC600, the values of Δ*G^θ^* were negative across the entire temperature range, and their absolute values increased with rising temperature, demonstrating that the adsorption process is spontaneous and that higher temperatures are more favorable for adsorption [86]. The positive value of Δ*H^θ^* indicates that the adsorption process is endothermic, and an increase in temperature can enhance adsorption. The positive value of Δ*S^θ^* suggests an increase in entropy during adsorption, implying greater freedom at the solid–liquid interface [39]. This may be attributed to the release of water molecules after TCH molecules are adsorbed onto the biochar surface, leading to an increase in system disorder [57].

#### 3.2.5. Adsorption Isotherms Analysis

The isotherm parameters determined at 25 °C are listed in Table 6, and the fitted curves are shown in Figure 10d. The Langmuir model assumes that adsorption involves monolayer coverage without interactions between adsorbed molecules. The Freundlich model describes non-ideal multilayer adsorption on heterogeneous surfaces with an exponential energy distribution [87]. The Temkin model is typically used to describe strong interactions, primarily electrostatic in nature, between the adsorbate and adsorbent [88]. Based on the correlation coefficients (R^2^) obtained from the isotherm model fitting for YBC600 and KYBC600, both the Langmuir and Temkin models adequately described the adsorption process of TCH onto the biochar. This indicates that TCH adsorption was influenced by multiple mechanisms collectively. For KYBC600, the Langmuir model yielded an R^2^ of 0.9881, signifying monolayer chemisorption of TCH. The Temkin model yielded an R^2^ of 0.9804, indicating the involvement of strong electrostatic interactions in the adsorption of TCH onto KYBC600.

According to the Langmuir model, the theoretical maximum adsorption capacities (*Q_m_*) at 298.15 K are 54.10 mg·g^−1^ for KYBC600 and 16.04 mg·g^−1^ for YBC600, representing a 3.37-fold enhancement upon KOH activation. The dimensionless separation factor *R_L_* gauges the favorability of Langmuir adsorption: 0 < *R_L_* < 1 indicates favorable uptake, *R_L_* > 1 unfavorable, *R_L_* = 1 linear, and *R_L_* < 0 irreversible [38]. At 298.15 K, both YBC600 and KYBC600 exhibit 0 < *R_L_* < 1 (Figure 10e), confirming that TCH adsorption on these biochars is thermodynamically favorable.

#### 3.2.6. Optimization of TCH Adsorption Conditions on KOH-Modified Biochar via RSM

To further optimize TCH adsorption on KYBC600 and investigate the synergistic effects of multiple factors on removal efficiency, Response Surface Methodology (RSM) was applied based on single-factor experiments. A Box–Behnken Design (BBD) generated by Design Expert 13 was used to optimize three parameters: adsorption time, biochar dosage, and initial solution pH. TCH adsorption efficiency (Y) was the response variable. Factor levels are given in Table 7, where the codes −1, 0, and 1 represent the low, middle, and high levels, respectively.

According to the experimental design, 17 sets of level experiments were conducted, with the results presented in Table 8. Analysis of variance (ANOVA) for the model was performed using Design-Expert 13 software (version 13.0.1), and the results are shown in Table 9.

The ANOVA results indicate that the selected model was highly significant (*p* < 0.0001), while the lack-of-fit term was not significant (*p* > 0.05). The difference between the adjusted determination coefficient ($R_{\mathrm{Adj}}^{2}$) and the predicted determination coefficient ($R_{\mathrm{Pred}}^{2}$) was less than 0.2. Furthermore, the precision value (38.2507) exceeded the standard threshold of 4. These results collectively demonstrate the model’s good fitting capability. Specifically, the adjusted determination coefficient $R_{\mathrm{Adj}}^{2}$ = 0.9837 indicates that the model explains 98.37% of the response variability. The coefficient of determination R^2^ = 0.9929 confirms a strong correlation between predicted and experimental values [89], validating the model’s suitability for analyzing and predicting TCH adsorption removal efficiency by KYBC600. Additionally, the model’s coefficient of variation (C.V. = 1.93% < 10%) indicates low dispersion between predicted and experimental values, confirming high model accuracy and experimental precision.

Comparison of the F-values allows the relative importance of each factor on TCH removal to be ranked [90]. According to Table 9, the linear term for biochar dosage (B) is highly significant (*p* < 0.0001) and possesses the largest F-value, indicating that dosage exerts the strongest influence on removal efficiency. Contact time (A) and initial pH (C) exhibit smaller F-values, reflecting a comparatively modest impact. Consequently, the factors can be ordered by decreasing influence as: biochar dosage (B) > initial pH (C) > contact time (A). Among the interaction terms, AC, B^2^, and C^2^ are all significant (*p* < 0.05), demonstrating that their combined effects also substantially affect TCH removal.

**Figure 11:**
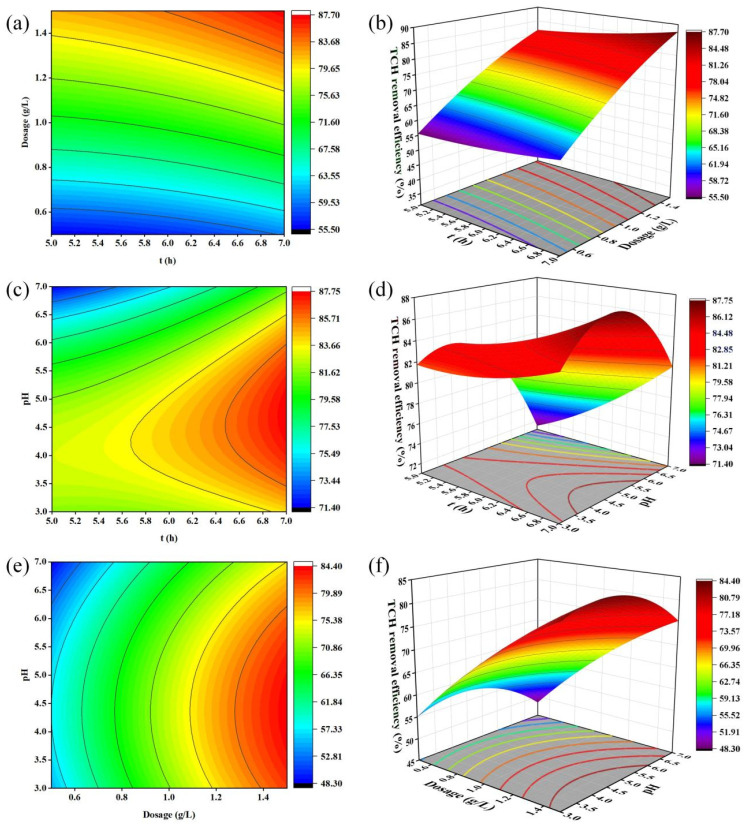
Contour plots (**a**,**c**,**e**) and response surface plots (**b**,**d**,**f**) showing the interactive effects of different variable pairs on tetracycline (TCH) removal efficiency: (**a**,b) adsorption time (A) and biochar dosage (B); (**c**,**d**) adsorption time (A) and initial solution pH (C); (**e**,**f**) biochar dosage (B) and initial solution pH (C). All experiments were conducted with an initial TCH concentration of 20 mg/L and a temperature of 25 °C. The elliptical nature of the contour plots indicates significant interactive effects between the corresponding variables.

Based on the regression equation generated by Design-Expert 13, three-dimensional response surfaces and two-dimensional contour plots were constructed with Origin 2021 to visualize the individual and interactive effects of contact time (A), KYBC600 dosage (B), and initial pH (C) on TCH removal (Y). The steepness of the response surface and the ellipticity of the contour lines indicate the magnitude of the interactive influence: a steeper surface with a larger slope and more oval contours signifies stronger interactions among the factors [91]. The response surfaces for the AB, AC, and BC interactions are presented in Figure 11. In all cases, the contour lines adopt markedly elliptical shapes, confirming that the pairwise interactions exert a pronounced effect on TCH removal.

The interactive effect of contact time (A) and biochar dosage (B) on TCH removal is illustrated in Figure 11a,b. Within the AB response surface, the contour lines are more densely packed along the dosage axis (B) than along the time axis (A), and the surface exhibits a steeper gradient along the B-axis. These features indicate that biochar dosage (B) exerts a substantially stronger influence on TCH removal efficiency than contact time (A).

The interactive influence of contact time (A) and initial pH (C) on TCH removal is depicted in Figure 11c,d. Within the AC response surface, the contour lines are more tightly spaced along the pH axis (C) than along the time axis (A), and the corresponding gradient is markedly steeper along the C-axis. These observations indicate that initial pH (C) exerts a more pronounced effect on TCH removal efficiency than contact time (A).

Similarly, Figure 11e,f illustrate the interaction between biochar dosage (B) and initial pH (C). In the BC response surface, the contour lines exhibit a higher density along the dosage axis (B) than along the pH axis (C), and the surface steepness is substantially greater for dosage. Consequently, biochar dosage (B) exerts greater influence than initial pH (C) in governing TCH removal.

Based on the experimental results in Table 8, a quadratic multiple regression equation was established relating adsorption time (A), biochar dosage (B), initial solution pH (C), and TCH adsorption rate (Y):

*Y* = 61.58 − 13.21*A* + 39.44*B* + 5.55*C* + 0.88*AB* + 0.92*AC* + 0.0033*BC* + 0.85*A*^2^ − 8.87*B*^2^ − 1.27*C*^2^

Optimization via response surface analysis predicted the maximum TCH removal (87.734%) to occur at an adsorption time of 7 h, a KYBC600 dosage of 1.5 g·L^−1^, and an initial pH of 4.709. For operational convenience, the pH was rounded to 5 while retaining the other two values. Under these adjusted conditions, three independent runs were conducted (Table 10), yielding an average TCH removal of 85.507%. The relative deviation between the predicted and observed values is only 2.54%, demonstrating excellent agreement and confirming the reliability of the model for predicting the process performance and optimizing TCH adsorption conditions.

#### 3.2.7. Adsorption–Desorption Regeneration Performance of KOH-Modified Biochar for TCH

**Figure 12:**
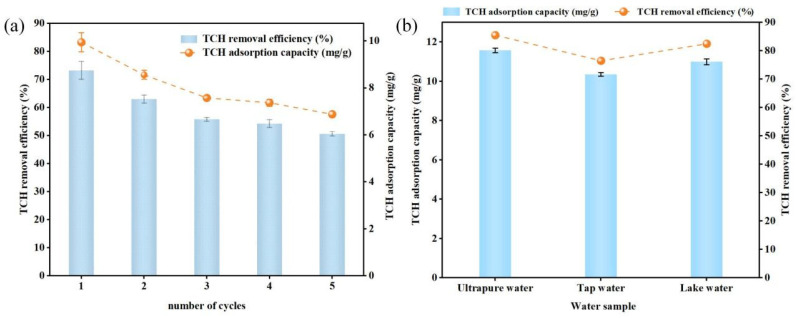
(**a**) Adsorption–desorption cycling performance of KYBC600 for TCH; (**b**) TCH removal efficiency in different water matrices after KYBC600 addition. Under the experimental conditions (6 h adsorption, 1.5 g/L dosage, 20 mg/L initial TCH, pH 5, 25 °C), the results confirmed that KYBC600 exhibited excellent reusability. Furthermore, it maintained a high TCH adsorption capacity across different aqueous matrices.

The recyclability of a biochar adsorbent is a key indicator of its environmental friendliness, cost-effectiveness, and long-term sustainability. Yu et al. investigated the reusability of ball-milled mulberry biochar for TCH adsorption through repeated washing with deionized water. They observed that after three adsorption–desorption cycles, the TCH adsorption capacity decreased to only one-third of the initial value [92]. Ultrasonic irradiation has been reported to effectively remove pollutants trapped in biochar pores, thereby facilitating regeneration [41]. Dai et al. adopted a regeneration strategy involving ultrasonic vibration followed by washing with deionized water. They reported that the TCH removal efficiency by biochar pyrolyzed at 500 °C decreased by 26.23% after three consecutive adsorption–desorption cycles [41]. As illustrated in Figure 12a, KYBC600 exhibits a gradual decline in both TCH removal efficiency and adsorption capacity upon successive cycles: the removal efficiency decreases from 73.24% to 50.64%, whereas the capacity drops from 9.95 mg·g^−1^ to 6.88 mg·g^−1^. This attenuation is attributed to incomplete TCH desorption during ultrapure-water ultrasonication, leaving residual TCH molecules partially occluding active sites on the biochar surface [93]. The TCH adsorption efficiency achieved in this study using the ultrasonic washing regeneration strategy aligns closely with the findings reported by Dai et al. [41]. This consistency further validates the feasibility of employing an ultrasonic washing approach for biochar recycling. The overall performance confirms that KYBC600 retains considerable regenerability and shows practical potential for cyclic TCH removal from aqueous systems.

Furthermore, it is important to note that although the biochar adsorption–ultrasonic washing regeneration cycle avoids the use of acidic or alkaline chemical reagents, it produces wastewater containing TCH. The effective treatment of this TCH-laden effluent is essential to ensure the environmental sustainability of this regeneration approach. Treatment methods such as biodegradation or advanced oxidation processes can be applied. Moreover, since regeneration relies solely on ultrasonic washing, the completeness of contaminant desorption must be carefully evaluated. Therefore, future research should focus on further investigating the irreversible adsorption of TCH onto biochar, which is crucial for achieving effective regeneration.

#### 3.2.8. Adsorption Efficacy of KOH-Modified Biochar for TCH in Different Water Matrices

Actual environmental water bodies typically contain various inorganic ions, organic acids, and other constituents that may influence the effectiveness of biochar in adsorbing TCH. Consequently, the removal efficiency of TCH by KYBC600 was comparatively investigated in three different water matrices: ultrapure water, tap water, and lake water. The results are presented in Figure 12b. After adding KYBC600, the adsorption removal efficiency of TCH reached 85.51% in ultrapure water, corresponding to an adsorption capacity of 11.58 mg·g^−1^. This performance was slightly superior to that observed in tap water and lake water. This difference is likely attributable to competitive adsorption occurring due to the numerous coexisting substances, such as organic matter, present in tap water and lake water [94]. Simultaneously, the removal efficiency of TCH in lake water was higher than in tap water. A possible explanation is that substances abundant in lake water, such as humic acid, played a beneficial role during the TCH adsorption process. The functional groups (e.g., hydroxyl, carboxyl) inherent to humic acid may engage in interactions such as hydrogen bonding with TCH molecules [95], thereby enhancing TCH adsorption.

Overall, KYBC600 maintains high TCH removal efficiency in ultrapure, tap, and lake water, demonstrating its strong adaptability to complex aqueous environments and highlighting its practical potential for TCH remediation in real water bodies.

### 3.3. Adsorption Mechanism of KOH-Modified Biochar for TCH in Water

#### 3.3.1. FTIR Analysis

**Figure 13:**
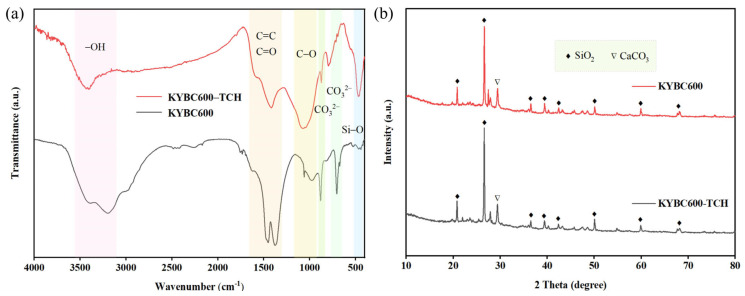
(**a**) FTIR spectra of KYBC600 before and after TCH adsorption; (**b**) XRD patterns of KYBC600 before and after TCH adsorption. All characterizations were performed at ambient temperature. FT-IR spectra were acquired over a range of 500–4000 cm^−1^ with a resolution of 4 cm^−1^ and 32 scans. XRD patterns were collected in the 2θ range of 5–85° using a step time of 0.5 s, an operating voltage of 30 kV, and a current of 20 mA. The spectral data suggested the formation of hydrogen bonding and π-π interactions during the adsorption of TCH onto KYBC600.

Figure 13a shows the evolution of FT-IR spectra of KYBC600 before and after tetracycline (TCH) adsorption. A pronounced alteration in the infrared absorption bands is observed after adsorption. Specifically, the intensity of the –OH stretching band is markedly attenuated, indicating that hydroxyl groups participate in the sorption process, most likely via hydrogen-bond formation with the –NH_2_ and –OH functionalities of TCH molecules [96,97]. The simultaneous decrease in the intensity of the C=C/C=O stretching bands signifies that these moieties are also implicated in TCH uptake. The conjugated enone structure of TCH can act as a strong π-electron acceptor, whereas the aromatic domains and the C=C/C=O functionalities of KYBC600 serve as π-electron donors, facilitating π–π stacking interactions [98] that enhance adsorption. Additionally, the –OH and C=O groups on the biochar surface can establish intermolecular hydrogen bonds with the C=O and –OH groups of TCH molecules [38]. After adsorption, the C–O stretching vibration exhibits a blue shift accompanied by an increased peak area, which can be attributed to π–π stacking interactions between the biochar and TCH molecules [99]. Furthermore, the diminished intensity of the ${\mathrm{CO}}_{3}^{2-}$ band suggests that carbonate species may also be involved in the adsorption process.

#### 3.3.2. XRD Analysis

Figure 13b shows the XRD patterns of KYBC600 before and after TCH adsorption. The primary phase composition of the biochar remained generally unchanged before and after adsorption, predominantly consisting of quartz (SiO_2_) and calcite (CaCO_3_). A weak graphite (002) diffraction peak at 2θ = 26.5° indicates the presence of a graphite-like microcrystalline structure in the biochar. In XRD patterns, a lower FOM value (figure of merit) corresponds to higher diffraction peak intensity, denoting greater concentration of the crystalline phase. After adsorption, the FOM value for CaCO_3_ increased from 6.9 to 12.0, suggesting decreased diffraction peak intensity. The initial solution pH during TCH adsorption was 5. The weakened CaCO_3_ peak intensity after adsorption may be attributed to gradual dissolution of CaCO_3_ present in KYBC600 during shaking under acidic conditions [100,101]. The released Ca^2+^ ions may form stable complexes with functional groups (e.g., phenolic hydroxyl, amino, and carbonyl groups) in TCH molecules via coordination bonds [102].

#### 3.3.3. SEM Analysis

**Figure 14:**
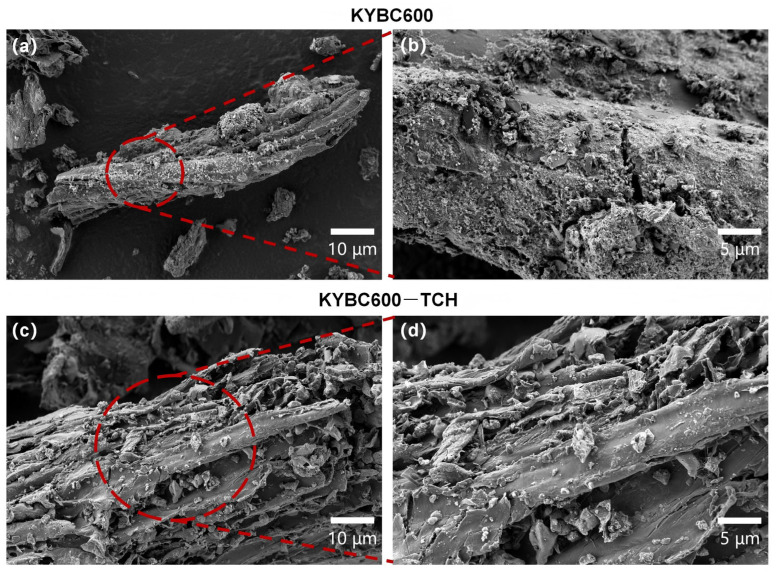
Scanning electron microscopy (SEM) images comparing the morphology of sample KYBC600 before and after tetracycline (TCH) adsorption. (**a**) Surface morphology of pristine KYBC600; (**b**) High-resolution view of the pristine KYBC600 surface; (**c**) Surface morphology of KYBC600 after TCH adsorption (KYBC600-TCH); (**d**) High-resolution view of the post-adsorption KYBC600-TCH surface. All images were acquired at an accelerating voltage of 2.0 kV. The micrographs revealed that the surface of KYBC600 became relatively smoother after TCH adsorption, with partial coverage of the original pores.

Figure 14 shows SEM images of KYBC600 before and after TCH adsorption at magnifications of 10 µm and 5 µm, respectively. Fine particles were observed on the biochar surface before adsorption. After adsorption, larger particles appeared, the surface became smoother, and most pores were clogged [103], indicating that TCH was adsorbed into the biochar pores. This demonstrates that pore filling is one of the mechanisms for TCH adsorption by KYBC600.

#### 3.3.4. XPS Analysis

**Figure 15:**
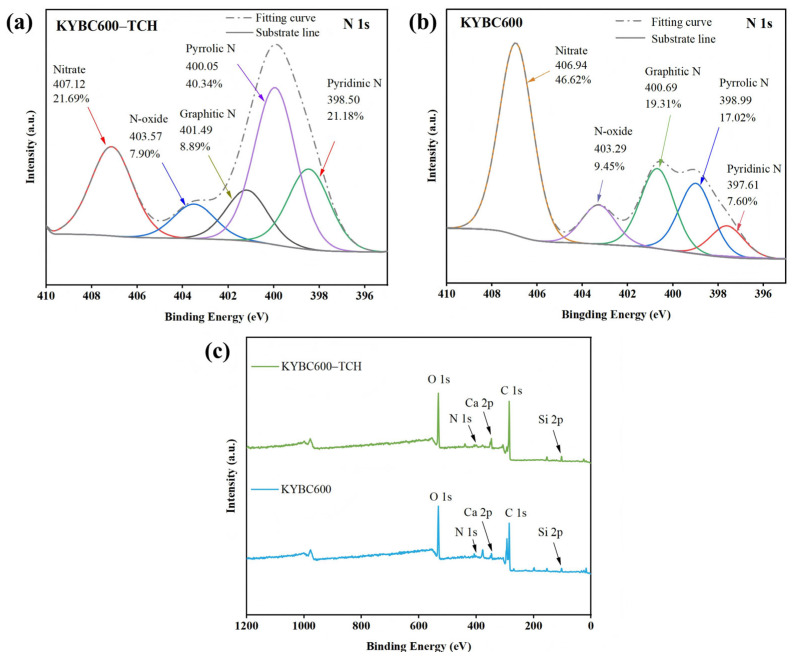
(**a**,**b**) N 1s and (**c**) XPS survey spectra of KYBC600 before and after TCH adsorption. Measurement conditions: Monochromatic Al Kα (1486.6 eV), 150 W, 650 μm spot, 14.8 kV, 1.6 A. Results indicate successful TCH adsorption and suggest Ca-TCH complexation.

**Figure 16:**
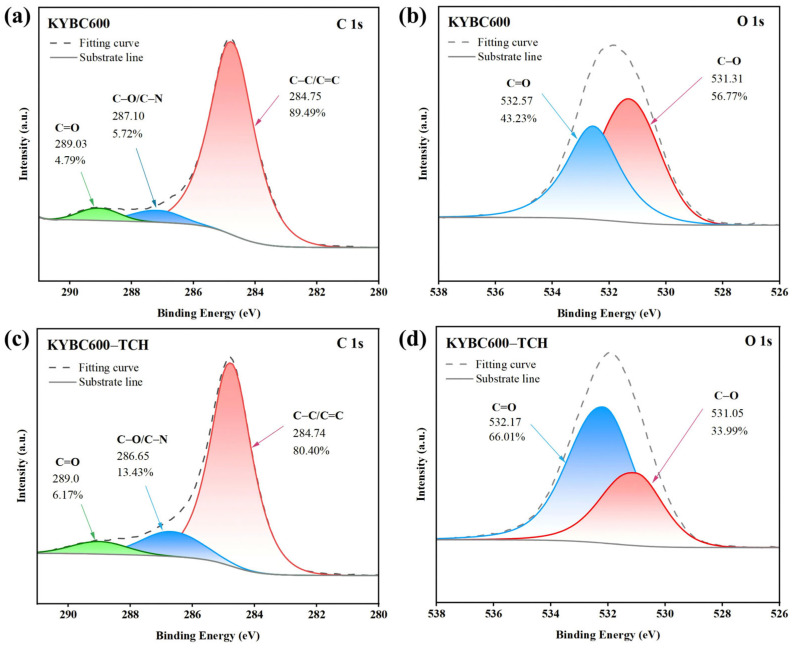
High-resolution (**a**,**c**) C 1s and (**b**,**d**) O 1s XPS spectra of KYBC600 before and after TCH adsorption. Measurement conditions: Monochromatic Al Kα (1486.6 eV), 150 W, 650 μm spot. Results indicate hydrogen bonding and π-π interactions between biochar and TCH.

XPS analysis was employed to further elucidate the adsorption mechanism. The XPS results of KYBC600 before and after TCH adsorption are presented in Figure 15 and Figure 16.

Following TCH adsorption, the signal intensities of C, N, and O elements on the KYBC600 surface increased (Figure 15c), confirming the successful loading of TCH. As shown in Figure 16a,c, the proportion of the C-C/C=C peak in KYBC600 decreased after TCH adsorption, which may be attributed to π-π interactions with the aromatic rings of TCH [104]. Concurrently, the proportions of C=O and C-O/C-N peaks increased, originating from the C-O, C-N, and C=O bonds within the TCH molecule. Furthermore, both the C-O/C-N and C=O peaks exhibited blue shifts to varying degrees after adsorption, suggesting the formation of hydrogen bonds between KYBC600 and TCH molecules [105].

In the N 1s spectra (Figure 15a,b), the characteristic peak at 400.05 eV showed a significant enhancement in intensity after TCH adsorption. This indicates interactions between nitrogen-containing groups (e.g., amide) in TCH and the active sites on KYBC600, further verifying the successful incorporation of TCH into the biochar. Additionally, the XPS survey spectrum (Figure 15c) revealed an enhanced Ca 2p peak intensity post-adsorption, possibly due to complexation between Ca species on the biochar surface and TCH molecules. This observation is consistent with the XRD characterization results.

#### 3.3.5. Proposed Adsorption Mechanism

In the single-factor experiments on pH effects, the solution pH influenced both the speciation and charge distribution of TCH molecules and the surface charge properties of the biochar. Meanwhile, the Temkin isotherm fitting confirmed the presence of strong electrostatic interactions between KYBC600 and TCH. Therefore, electrostatic interactions represent a primary adsorption mechanism.

**Figure 17:**
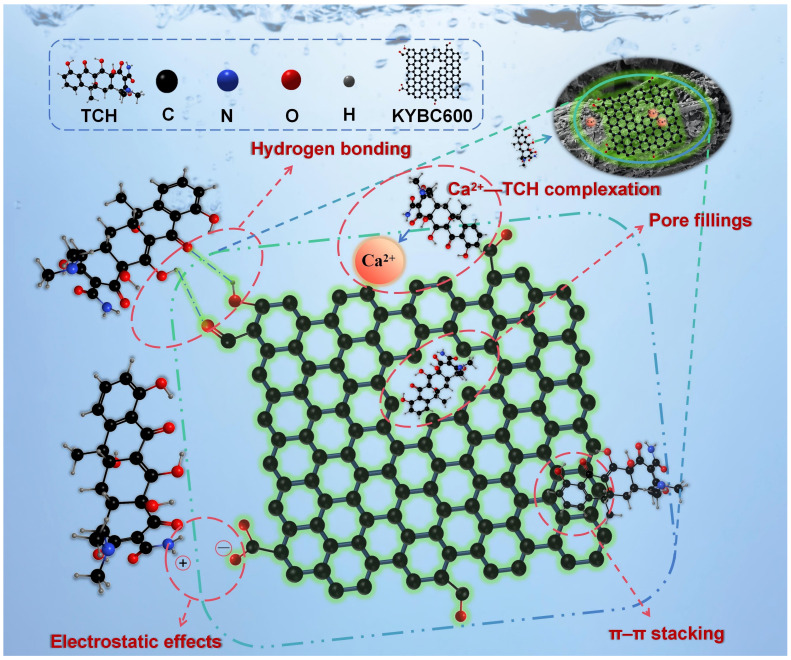
Possible adsorption mechanism of KYBC600 for TCH in water. The proposed adsorption mechanism of TCH onto KYBC600 involves pore filling, hydrogen bonding, electrostatic interactions, π-π interactions, and complexation.

A comprehensive analysis of FTIR, XRD, and SEM results before and after TCH adsorption, combined with pH-dependent experiments, indicates that the adsorption of TCH onto KOH-modified biochar (KYBC600) involves multiple mechanisms: pore filling, electrostatic interactions, π–π stacking, Ca^2+^–TCH complexation, and hydrogen bonding, as illustrated in Figure 17.

### 3.4. Comparative Analysis with Other Adsorbents

This study enhanced the adsorption efficacy of biochar for TCH through a modification method involving KOH impregnation followed by pyrolysis. Table 11 compares the maximum adsorption capacities and mechanisms of various biochar-based adsorbents for tetracycline hydrochloride (TCH) in aqueous solution. The KOH-modified biochar (KYBC600) prepared in this study exhibited a higher maximum adsorption capacity for TCH than most reported biochar-based adsorbents, demonstrating its favorable adsorption performance.

## 4. Conclusions and Outlook

In this study, biochar derived from yak manure was prepared through high-temperature pyrolysis combined with KOH modification, exhibiting a developed pore structure and abundant surface functional groups. Single-factor experiments and response surface methodology (RSM) optimization showed that the KOH-modified biochar (KYBC600) achieved a TCH adsorption rate of 85.51% and an adsorption capacity of 11.58 mg·g^−1^ under optimized conditions. The Langmuir model indicated a theoretical maximum adsorption capacity (*Q_m_*) of 54.10 mg·g^−1^ for KYBC600 at 298.15 K, which exceeds that of most reported adsorbents. The adsorption of TCH onto KYBC600 was determined to be a spontaneous, endothermic, and entropy-driven process, consistent with monolayer chemisorption accompanied by strong electrostatic interactions. The process involved film diffusion, intraparticle diffusion, and eventual adsorption–desorption equilibrium. KYBC600 retained a TCH adsorption rate of 50.64% after five adsorption–desorption cycles and exhibited effective TCH removal in various real water matrices. Based on FTIR, SEM, and XRD analyses, the key mechanisms of TCH adsorption onto KYBC600 were identified as pore filling, electrostatic interactions, π–π stacking, hydrogen bonding, and Ca^2+^–TCH complexation. These results provide valuable reference for the resource utilization of yak manure and the remediation of low-concentration TCH-contaminated wastewater.

The KOH-modified biochar developed in this study exhibits promising performance in adsorbing tetracycline hydrochloride (TCH) from aqueous solutions. Despite this, several limitations and challenges remain concerning the selection of modification methods, recyclability, and practical wastewater treatment.

Regarding modification methods, although KOH activation is widely used to enhance the specific surface area and porosity of biochar, the use of this strong alkali requires careful consideration of potential environmental risks and the long-term stability of the resulting material. Future studies should therefore explore milder and more efficient modification strategies to improve both the adsorption performance and stability of biochar, while also developing a simpler preparation and activation protocol.

In terms of recyclability, this study employed an ultrasonic washing regeneration strategy. Although the biochar retained a relatively high TCH adsorption capacity after five adsorption–desorption cycles, this does not fully elucidate the scientific mechanism behind its irreversible adsorption behavior. Subsequent work will further investigate its recycling potential and assess the economic feasibility for practical application.

Concerning real-world wastewater treatment, most current studies, including this one, focus on laboratory-scale simulated wastewater. Actual wastewater has a significantly more complex composition and variable contaminant concentrations. Therefore, future research should prioritize application studies within real domestic wastewater systems, with comprehensive evaluation of both economic and environmental benefits. This work aims to provide more reliable technical support for the resource utilization of yak manure and the treatment of low-concentration TCH-contaminated wastewater.

## Figures and Tables

**Table 1:** The BET specific surface area, pore structure parameters, and yield of yak manure-derived biochar prepared at different pyrolysis temperatures are presented. The BET measurements were conducted using nitrogen and helium gases under liquid nitrogen conditions, following a 30 min pre-treatment of the samples in an oven at 120 °C. The results indicate that YBC600 exhibits a relatively large specific surface area.

Biochar	Specific Surface Area (m^2^·g^−1^)	Pore Volume (cm^3^·g^−1^)	Average Pore Diameter (nm)	Yield (%)
YBC500	8.69	0.022	10.17	52.05%
YBC600	26.65	0.042	6.38	51.99%
YBC700	16.64	0.081	19.59	51.90%

**Table 2:** BET results of biochar before and after KOH modification. The BET measurements were performed using nitrogen and helium gases under liquid nitrogen conditions (77 K), with samples pre-degassed at 120 °C for 30 min prior to analysis. The results indicate that KOH modification effectively enhanced the specific surface area and pore volume of the biochar, leading to an improved pore structure.

Biochar	Specific Surface Area (m^2^·g^−1^)	Pore Volume (cm^3^·g^−1^)	Average Pore Diameter (nm)
YBC600	26.65	0.042	6.38
KYBC600	96.06	0.101	4.21

**Table 3:** Adsorption kinetic parameters of biochar for TCH. The adsorption kinetics experiments were conducted under the following conditions: a temperature of 25 °C, an initial TCH concentration of 20 mg/L, an initial solution pH of 5, a biochar dosage of 0.5 g/L, and a contact time ranging from 0 to 360 min. The results revealed that the adsorption process of TCH onto both KYBC600 and YBC600 was better described by the pseudo-second-order kinetic model.

Model	Parameter	Biochar	
		YBC600	KYBC600
Pseudo-first-order kinetics	*Q_e_*/(mg·g^−1^)	11.0468	19.3669
	*k*_1_/(min^−1^)	0.0361	0.8812
	*R* ^2^	0.8920	0.9519
Pseudo-second-order kinetics	Q_e_/(mg·g^−1^)	12.0069	19.7931
	*k*_2_/(g·mg^−1^·min^−1^)	0.0048	0.0586
	*R* ^2^	0.9369	0.9765

**Table 4:** Intraparticle diffusion model parameters for TCH adsorption by biochar. The adsorption experiments were conducted under the following conditions: a temperature of 25 °C, an initial TCH concentration of 20 mg/L, an initial pH of 5, a biochar dosage of 0.5 g/L, and a contact time ranging from 0 to 360 min. The results demonstrated that the TCH adsorption process involved three consecutive stages: film diffusion, intra-particle diffusion, and finally, adsorption–desorption equilibrium.

Parameter	Biochar	
	YBC600	KYBC600
*k_i_*_1_/(mg·(g·min^1/2^)^−1^)	1.2293	1.9435
*C*_1_	1.2002	9.5926
*R*_1_^2^	0.7442	0.9699
*k*_i2_/(mg·(g·min^1/2^)^−1^)	0.7737	0.2296
*C*_2_	2.4690	17.3574
*R*_2_^2^	0.7886	0.5666
*k*_i3_/(mg·(g·min^1/2^)^−1^)	0.1743	0.1654
*C*_3_	8.6101	17.594
*R*_3_^2^	0.9689	0.6798

**Table 5:** Adsorption thermodynamic parameters of TCH on biochar. The adsorption experiments were conducted at temperatures of 15, 25, and 35 °C with an adsorption time of 6 h, a biochar dosage of 0.5 g/L, an initial TCH concentration of 20 mg/L, and an initial solution pH of 5. The thermodynamic analysis revealed that the adsorption of TCH onto KYBC600 was a spontaneous, endothermic, and entropy-increasing process. Furthermore, KOH modification was found to be more favorable for the adsorption of TCH onto the biochar.

Biochar	Temperature T/(K)	∆G^θ^/(kJ·mol^−1^)	∆H^θ^/(kJ·mol^−1^)	∆S^θ^/(J/mol^−1^·K^−1^)
YBC600	288.15	−2.15	18.24	70.85
	298.15	−2.87		
	308.15	−3.59		
KYBC600	288.15	−4.02	25.67	103.12
	298.15	−4.92		
	308.15	−5.81		

**Table 6:** Adsorption isotherm parameters of TCH on biochar. The experiments were conducted under controlled conditions: temperature of 25 °C, adsorption time of 6 h, biochar dosage of 0.5 g/L, initial TCH concentration of 20 mg/L, and initial solution pH of 5. The results demonstrated that the adsorption of TCH onto the biochar primarily followed monolayer chemical adsorption with significant electrostatic interactions. The modified KYBC600 surface was particularly favorable for TCH attachment due to its enhanced surface properties.

Model	Parameter	Biochar	
		YBC600	KYBC600
Langmuir Model	*Q_m_*/(mg·g^−1^)	16.0424	54.1034
	*K_L_*_/_(L·mg^−1^)	0.0824	0.0420
	*R* ^2^	0.9381	0.9881
Freundlich Model	*K*_F/_(mg·g^−1^)(mg·L^−1^)^−1/n^	3.5525	6.2062
	1/*n*	3.2072	2.3116
	*R* ^2^	0.8702	0.9438
Temkin Model	*K*_T_/(L·g^−1^)	0.8522	0.4032
	*b*	741.9726	207.7169
	*R* ^2^	0.9124	0.9804

**Table 7:** Factors, levels, and codes for the Design Expert experimental design. The experiments were conducted with an initial TCH concentration of 20 mg/L and at a temperature of 25 °C.

Factor	Code	Coded Level		
		−1	0	1
Adsorption time/(h)	*A*	5	6	7
Biochar dosage/(g·L^−1^)	*B*	0.5	1	1.5
Initial solution pH	*C*	3	5	7

**Table 8:** Experimental design and results of the BBD model. The experiments were conducted with an initial TCH concentration of 20 mg/L and at a temperature of 25 °C.

Code	A—Adsorption Time	B—Biochar Dosage	C—Initial Solution pH	TCH Adsorption Rate(%)
1	0	1	−1	87.66
2	−1	1	0	82.99
3	0	0	0	73.53
4	0	0	0	72.24
5	1	0	−1	73.01
6	0	1	1	74.84
7	1	0	1	70.69
8	0	0	0	71.37
9	0	−1	1	48.37
10	−1	0	1	59.84
11	0	−1	−1	55.57
12	1	−1	0	58.44
13	1	1	0	86.86
14	0	0	0	72.94
15	0	0	0	72.50
16	−1	−1	0	56.33
17	−1	0	−1	69.55

**Table 9:** ANOVA results for response surface methodology data. Under the experimental conditions of an initial TCH concentration of 20 mg/L and a temperature of 25 °C, the model demonstrated a good fit. The results revealed that the order of influence of the three examined factors on the TCH removal efficiency was as follows: biochar dosage (B) > initial solution pH (C) > adsorption time (A).

Source	Sum of Squares	df	Mean Square	*F*-Value	*p*-Value	Significance
Model	1747.95	9	194.22	108.56	<0.0001	Significant
*A*	51.53	1	51.53	28.80	0.0010	-
*B*	1458.34	1	1458.34	898.24	<0.0001	-
*C*	87.30	1	87.30	48.80	0.0002	-
*AB*	0.7765	1	0.7765	0.4340	0.5311	-
*AC*	13.66	1	13.66	7.63	0.0280	-
*BC*	0.0000	1	0.0000	0.0000	0.9962	-
*A* ^2^	3.07	1	3.07	1.72	0.2315	-
*B* ^2^	20.70	1	20.70	11.57	0.0114	-
*C* ^2^	109.41	1	109.41	61.15	0.0001	-
Residual	12.52	7	1.79	-	-	-
Lack of fit	9.94	3	3.31	5.14	0.0738	Not significant
Pure error	2.58	4	0.6448	-	-	-
Total	1760.47	16	-	-	-	-
*R*^2^ = 0.9929, $R_{\mathrm{Adj}}^{2}$ = 0.9837, $R_{\mathrm{Pred}}^{2}$ = 0.9073, C.V. = 1.93%, Precision = 38.2507						

Note: *p* < 0.001: Extremely significant; *p* < 0.05: Significant; *p* > 0.05: Not significant.

**Table 10:** Validation results under optimal adsorption conditions.

Optimal Adsorption Conditions			Actual TCH Removal Rate(%)			
Adsorption Time(h)	Biochar Dosage(g·L^−1^)	Initial Solution pH	1	2	3	Mean Value
7	1.5	4.709	86.493	85.136	84.891	85.507

Note: The validation experiments were conducted under the following fixed conditions: adsorption time of 7 h, biochar dosage of 1.5 g/L, initial solution pH of 5, temperature of 25 °C, and initial TCH concentration of 20 mg/L. The results demonstrate a close agreement between the model-predicted values and the experimental data, confirming the model’s reliability.

**Table 11:** Comparison of TCH Adsorption by Biochar in Aqueous Solution. Comparative analysis reveals that the TCH adsorption performance of KYBC600 is superior to that of most reported adsorbents.

Feedstock	Maximum Adsorption Capacity (mg·g^−1^)	Actual Removal Capacity/Rate (Reported Conditions) (mg·g^−1^)	TCH Residual Concentration/(mg/L)	Primary Adsorption Mechanism	References
Wood Ear Residue	11.90	4.13	1.26	π-π electron donor-acceptor interaction	[41]
Shiitake Spent	37.95	~22.14	77.86	Pore filling, π-π interaction, complexation, and hydrogen bonding	[81]
Grapefruit Peel	34.58	37.92	2.6	Pore filling, charge interaction, and chemical bonding	[106]
Wheat Straw	55.23	31.48	11.85	Pore filling, hydrogen bonding, π-π interaction, and electrostatic interaction	[107]
Rice Straw	50.72	~96% Removal	-	π-π interaction and electrostatic interaction	[108]
Rice Husk	46.95	39.36	60.64	Hydrogen bonding, hydrophobic interaction, and π-π interaction	[109]
Corn Stalk	33.53	17.66	2.34	Hydrogen bonding and π-π interaction	[110]
AA Residue	42.31	~40.1	-	Hydrogen bonding interaction	[111]
Microalgae	42.7	~90% Removal	-	Physical adsorption (attributed to enhanced specific surface area and improved pore structure), coupled with the presence of nitrogen elements	[112]
Waste Fiberboard	6.37	5.49	6.28	π-π interaction	[113]
Cattle Manure	5.38	2.25	0.99	Hydrophobic interaction and π-π electron donor-acceptor interaction	[114]
Yak Manure	54.10	~85.51% Removal	2.94	Pore filling, electrostatic interaction, π–π interaction, hydrogen bonding, and Ca^2+^–TCH complexation	This study

## Data Availability

Data are contained within the article.
